# Redox-modulated ERK dynamics support wound-dependent tissue formation during early planarian regeneration

**DOI:** 10.1016/j.isci.2026.115640

**Published:** 2026-04-08

**Authors:** Martijn Heleven, Maria Dolores Molina, Vincent Jaenen, Karolien Bijnens, Francesc Cebrià, Karen Smeets

**Affiliations:** 1 Centre for Environmental Sciences, Zoology: Biodiversity and Toxicology, Hasselt University, Diepenbeek, Belgium; 2 Department of Bioengineering, IQS School of Engineering, University of Ramon Llull, Barcelona, Catalonia, Spain; 3 Department of Genetics, Microbiology and Statistics, Faculty of Biology, University of Barcelona, Barcelona, Catalonia, Spain; 4 Institute of Biomedicine of the University of Barcelona (IBUB), University of Barcelona, Barcelona, Catalonia, Spain

**Keywords:** Histology, Biological sciences, Molecular biology, Cell biology

## Abstract

The mitogen-activated protein kinase-extracellular signal-regulated kinase (MAPK-ERK) pathway is essential during regeneration as it guides stem cell proliferation, differentiation, and survival. We identified redox-dependent components of MAPK-ERK signaling whose coordinated activity is required for full-body regeneration and patterning. Wound orientation was found to influence ERK activation and redox dynamics, with anterior-facing wounds showing elevated ERK activity 3–6 h post-amputation, together with increased superoxide and hydrogen peroxide levels, compared to posterior-facing wounds. Disruption of MAPK signaling produced distinct effects on anterior versus posterior regeneration that depended on both wound orientation and the fragment’s original anterior-posterior identity. In fragments containing anterior- and posterior-facing wounds, this molecular gradient was re-established within 6 h. Additionally, our findings suggest a feedback regulatory circuit controlling ERK activation, in which the transcription factor egr-4 (early growth response protein 4) inhibits MAPK phosphatase activity. In summary, amputation-induced redox signals coordinate with the MAPK-ERK-egr4 signaling pathway to ensure correct regeneration and tissue patterning in planarians.

## Highlights

• Anterior wounds show higher ROS and ERK activity than posterior wounds within 3–6 h
• egr-4 regulates ERK activity by inhibiting MAPK phosphatase activity
• β-catenin RNAi rescues anterior regeneration defects caused by MAPK inhibition

## Graphical abstract

**Figure:**
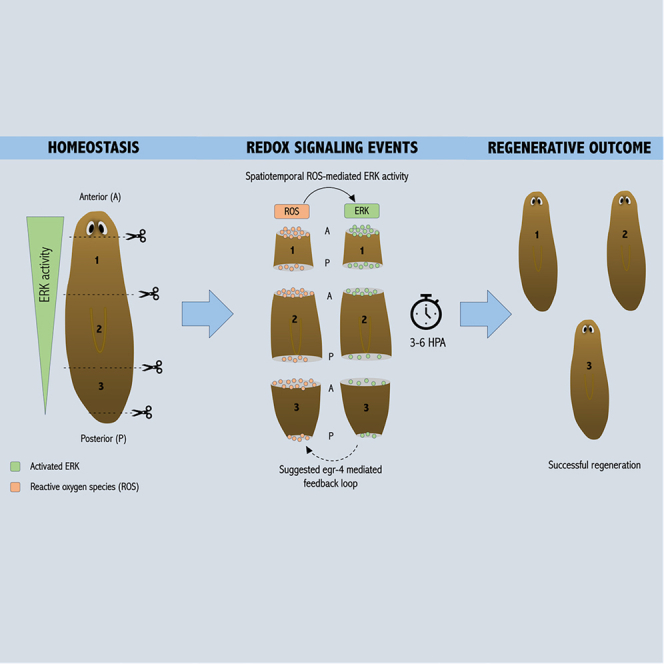

## Introduction

Regeneration is a complex biological phenomenon driven by mature cell dedifferentiation or the self-renewal capacity of adult pluripotent stem cells, enabling tissue repair and organ morphogenesis.^1^ The ability to regenerate is a widespread yet highly variable trait within the animal kingdom.^2^^,^^3^ Most vertebrates, including humans, possess minimal regenerative capacity throughout their adult life.^4^ In contrast, the freshwater planarian *Schmidtea mediterranea* is able to regenerate any missing body part, including the central nervous system and digestive tract, highlighting its exceptional plasticity and regenerative capabilities. Approximately 20% of the planarian body consists of adult pluripotent stem cells, known as neoblasts, which migrate, proliferate, and eventually differentiate to replace damaged or lost tissue.^5^^,^^6^^,^^7^^,^^8^ Therefore, these highly regenerative animals allow us to study the signaling events that regulate cell behavior and communication during tissue regeneration.^9^

A fundamental aspect of this regenerative capability is the precise patterning of the new tissues, which entails the re-establishment of the different body axes, e.g., dorsoventral (D-V) axis and anterior-posterior (A-P) axis, which define the body’s dorsal-to-ventral and head-to-tail orientation, respectively. Dozens of cell types and tissues are distributed along these axes, requiring neoblasts to recognize and replace missing cells and tissues in various regenerative contexts. After wounding, positional control genes (PCGs) direct correct tissue patterning.^10^^,^^11^ Previous research has identified several key signaling components of conserved regulatory networks in planarians. For example, bone morphogenic protein (BMP) primarily regulates D-V patterning, while Wingless/int-1 (Wnt) and Hedgehog (Hh) are crucial for re-establishing the A-P axis.^12^^,^^13^^,^^14^^,^^15^^,^^16^^,^^17^ These genes are primarily expressed in subepidermal muscle cells, which serve as a positional coordinate system during planarian regeneration.^18^

In response to injury, polarity determinants are rapidly induced in muscle cells to re-establish PCG expression based on the amputation site relative to the body axes.^18^ PCGs, such as *Wnt1*, provide positional information to neighboring neoblasts, guiding their differentiation into the missing tissues.^19^^,^^20^ *Wnt1* expression is rapidly increased at the wound site, activating β-catenin predominantly in the posterior region, with a decreasing trend along the A-P axis.^12^^,^^21^ While Wnt/β-catenin signaling specifies posterior identity, anterior regeneration is promoted by a distinct set of factors, including the Wnt inhibitor notum, the activin antagonist follistatin, and the transcription factors runt-1 and early growth response protein 4 (egr-4), among others.^22^^,^^23^^,^^24^^,^^25^ These anterior determinants are expressed in a polarized manner and depend strongly on early extracellular signal-regulated kinase (ERK) activity, suggesting a key role for MAPK-ERK signaling in initiating anterior specification,^26^^,^^27^ potentially by interacting with the Wnt/β-catenin pathway.^28^^,^^29^

The evolutionarily conserved MAPK pathway encompasses a family of proline-serine/threonine kinases, which play crucial roles in the consecutive transduction of signals, from the cell membrane to the nucleus, and can be initiated by both extracellular and intracellular stimuli.^30^ Within the animal kingdom, three types of MAPK proteins have been identified: ERKs, the c-Jun N-terminal kinases (JNKs), and the p38 kinases. Their activity alters the expression of multiple genes involved in cellular proliferation, differentiation, and apoptosis.^31^ The upstream activator of ERK, i.e., MAPK/ERK kinase (MEK), was found to be important for anteriorization in planarians, as the pharmacological inhibition of MEK activity completely blocks the anterior regeneration process, leaving regenerating tails in a dormant state.^27^ ERK activation occurs via an epidermal growth factor receptor (EGFR), and this signaling event is necessary for proper neoblast differentiation and morphogenesis during regeneration and homeostasis.^32^^,^^33^ Additionally, research by Owlarn and colleagues determined that ERK activation is induced during early regeneration in a stem cell-independent manner. They reported that ERK activation, appearing to be the most upstream initiator of planarian regeneration, occurs via injury signals not originating from newly synthesized proteins.^27^

We recently identified reactive oxygen species (ROS) as a possible activator of the MAPK-ERK pathway during the initial phases of regeneration.^34^ After MEK inhibition, the planarian regenerating fragments remain in a dormant state until a new wound is inflicted, reactivating the process of regeneration in an ROS-dependent manner.^34^^,^^35^ It is generally accepted that the catalytic cysteine residues, present on cytokine receptors and growth factors upstream of the pathway as well as downstream signaling molecules like MAPK3Ks, can serve as ROS-activating sites.^31^^,^^36^^,^^37^ After inducing planarian regeneration via amputation, an ROS burst was observed at both the anterior and posterior wound sites.^38^ Interfering with this ROS production affects stem cell differentiation but not their migration and proliferation. Additionally, inhibition of ROS production affects proper patterning and polarity.^38^

This study aimed to (1) determine whether ERK activation in *S. mediterranea* depends on the location and orientation of the wound along the A-P axis, (2) pinpoint the specific time at which differences in pERK levels are established, (3) investigate whether early pERK gradient formation is orchestrated upstream by redox signals and identify potential feedback mechanisms, and (4) explore if the MAPK-ERK signaling cascade potentially counteracts the Wnt/β-catenin signaling events.

## Results

### MAPK-ERK signaling is essential for regeneration

**Figure 1:**
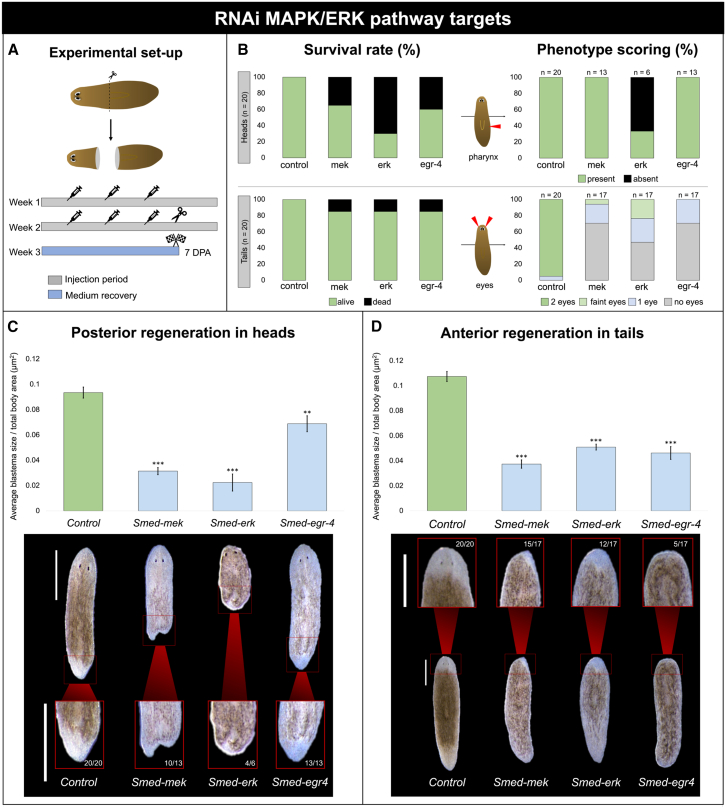
RNAi-induced KD of MAPK-ERK-related genes strongly affects regeneration (A) Graphical representation of the experimental setup. RNAi-treated animals were transversally amputated above the pharynx to generate head and tail fragments (*n* = 20), which were allowed to regenerate for 7 DPA. (B) Survival rates (%) of head and tail fragments and phenotype scoring (pharynx presence in heads and eye development in tails are shown). Color code is as follows: “survival rate”: green, alive; black, death; “pharynx presence”: green, present; black, absent; “eye development”: green, 2 eyes; light green, fainted eyes; blue, 1 eye; gray, no eyes. Note that “1 eye” scoring following *Smed-erk* KD corresponds to cyclopia formation (Figure S1). (C and D) Posterior blastema formation in head fragments (C) and anterior blastema formation in tail fragments (C). The graphs represent the differences in average blastema size relative to the full body size (μm^2^) after KD of the target genes (7 DPA). The corresponding phenotypes are shown below. Color code is as follows: green, control; blue, RNAi-treated. Scale bars are 500 μm. Data represent the mean ± SEM from two independent experiments. Statistical significance was assessed using one-way ANOVA. ∗, *p* < 0.05; ∗∗, *p* < 0.01; ∗∗∗, *p* < 0.001.

Knockdown (KD) of *Smed-mek* (mitogen-activated protein kinase kinase), *Smed-erk* (mitogen-activated protein kinase), and *Smed-egr-4* (early growth response protein 4) led to regenerative defects in both heads and tails, affecting blastema formation and eye development at 7 days post amputation (DPA) (Figures 1 and S1). The survival rate (%) of the heads was severely affected after *Smed-mek* (65%), *Smed-erk* (35%), and *Smed-egr-4* (65%) KDs. In the regenerating heads that survived the *Smed-mek* KD, a clearly bifurcated, newly formed tail could be observed (77%) (Figures 1C and S1). Additionally, head regression was observed after *Smed-erk* KD (67%), which co-occurred with absence of the pharynx (Figures 1B, 1C, and S1). Tail survival rate (%) was only mildly reduced after each KD (15%). Eye development was absent after *Smed-mek* (71%), *Smed-erk* (47%), and *Smed-egr-4* (70%) KDs (Figures 1B, 1D, and S1). In cases where tails regenerated eyes, development was impaired, resulting in one eye after *Smed-mek* (24%) and *Smed-egr-4* (29%) KDs, whereas cyclopia formation was observed after *Smed-erk* KD (29%) (Figure S1).

Moreover, KD of all these targets significantly impaired regeneration compared with the control group (Figures 1C, 1D, and S1). Here, “anterior regeneration” refers to tails regenerating a new head, and “posterior regeneration” refers to heads regenerating a new tail. Anterior regeneration was significantly more impaired than posterior regeneration after *Smed-egr-4* KD, while the inverse was true after *Smed-erk* KD. No differences in the effects on anterior and posterior regeneration could be observed after *Smed-mek* KD (Table S1).

### MEK inhibition induces wound- and position-dependent regenerative defects

#### MEK inhibition induces A-P axis-dependent regenerative defects

**Figure 2:**
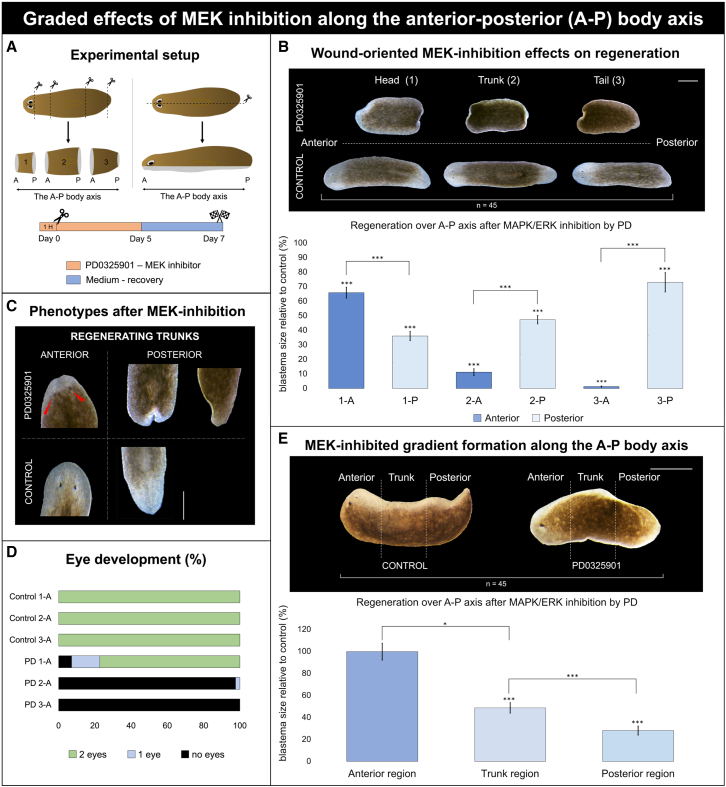
Effects on regeneration along the A-P axis after MEK inhibition (A) Graphical representation of the experimental setup. The animals were cut along the sagittal plane (*n* = 32) or transversely into three fragments (*n* = 48), eliminating the tips of both head and tail fragments, generating three fragments (1, 2, 3), each with anterior (A)- and posterior (P)-oriented wound sites. MEK inhibition was carried out via PD0325901 treatment (orange), administered 1 h prior to amputation and continued for 5 days during regeneration, after which the animals were placed in fresh culture medium (blue). Regenerative outcomes were measured at 7 DPA, including relative blastema size and eye development. (B) Effects of MEK inhibition on regeneration along the A-P axis. Representative control and MEK inhibition-induced phenotypes of the three fragments at 7 DPA following transversal amputation are shown. The graph displays quantified anterior (dark blue) and posterior (light blue) blastema sizes of all three fragments: fragment 1 (1-A and 1-P), fragment 2 (2-A and 2-P), and fragment 3 (3-A and 3-P), all relative to their corresponding control group (%). Scale bars are 500 μm. Data represent the mean ± SEM from three independent experiments. Statistical significance was assessed using one-way ANOVA. ∗, *p* < 0.05; ∗∗, *p* < 0.01; ∗∗∗, *p* < 0.001. (C) Anterior and posterior blastema phenotypes after MEK inhibition and in controls at 7 DPA. Red arrowheads indicate the presence of newly formed eyes in existing tissue. Scale bars are 500 μm. (D) Eye development in anterior blastemas at 7 DPA of all three tissue fragments shown in (B). Color code: green, two eyes; blue, 1 eye; black, no eyes. (E) MEK inhibition-induced gradient formation along the A-P axis, Representative control and MEK inhibition-induced phenotypes at 7 DPA following sagittal amputation are shown. The graph displays quantified anterior (dark blue) and posterior (light blue) blastema sizes of all three regions: posterior, trunk, and anterior, all relative to their corresponding control group (%). Scale bars are 500 μm. Data represent the mean ± SEM from three independent experiments. Statistical significance was assessed using one-way ANOVA. ∗, *p* < 0.05; ∗∗, *p* < 0.01; ∗∗∗, *p* < 0.001.

To further investigate the A-P differences in MEK inhibition, we used a quantitative approach comparing the effect on anterior and posterior regeneration within fragments, and between fragments originating from different locations along the original A-P axis. Following removal of the head and tail tips, the animals were amputated to generate three tissue fragments with both anterior and posterior wounds (Figure 2A).

MEK inhibition significantly affected both anterior and posterior regeneration, compared to the control group, in all tissue fragments (Figure 2B). In the tail and trunk fragments, anterior regeneration was more severely affected than posterior regeneration after MEK inhibition (Figure 2B). Although regeneration often initiated in posterior-oriented wounds of trunk fragments, the blastemas were frequently bifurcated or malformed (Figure 2C). The opposite was observed in the regenerating fragments originating from the most anterior position, the original head (Figure 2B, fragment 1). Here, posterior regeneration was significantly more blocked than anterior regeneration. Comparing the effects on wounds with the same polarity across different body fragments revealed that the degree to which regeneration was impaired depended on the fragment’s original position along the A-P axis of the intact animal (Figure 2B). Specifically, anterior blastema regeneration was significantly more reduced in fragments derived from the most posterior region of the original A-P axis, and vice versa for posterior regeneration. When comparing anterior blastemas of the different fragments, we observed that the regenerative defects were significantly less severe in the most anterior fragment (1-A) and progressively increased in fragments derived from more posterior positions along the original A–P axis (2-A and 3-A), with fragment 3-A showing the strongest impairment. In accordance, photoreceptor development was more impaired in posterior fragments (2-A and 3-A): almost none formed eyes, whereas 80% of the most anterior fragment (1A) produced two photoreceptors (Figures 2B and 2D). When eyes formed in MEK inhibition-induced trunks, they arose within or near pre-existing tissue, in contrast to controls, where eyes developed in the newly formed tissue (Figures 2B and 2C; red arrowheads).

In an additional experiment, we observed that the extent to which anterior regeneration was affected by MEK inhibition depends on the axial identity of the anterior-facing wound (Figure S2). Following 5 days of MEK inhibition and subsequent washout, fragment 1A, in which the anterior-facing wound is derived from head-proximal tissue, largely recovered blastema, eye, and anterior neural regeneration. In contrast, 2A fragment, whose anterior-facing wound originated from more posterior tissue, failed to recover and retained severe anterior defects at 14 DPA (Figure S2B). Continuous PD exposure abolished regeneration in both fragment types, confirming that recovery requires drug removal (Figure S2C).

### Region-specific sensitivity along the intact A-P axis following MEK inhibition

To explore how MEK inhibition affects regeneration in the context of an intact A-P axis, animals were sagittally amputated, which created a continuous tissue fragment with a longitudinal wound (Figure 2E). Regeneration was differentially affected along the longitudinal wound. No significant effects on blastema size were observed in the head region, while regeneration in the trunk and tail regions was significantly impaired compared with the controls. Notably, the strongest reduction in blastema size following MEK inhibition occurred in the tail region.

In summary, MEK inhibition resulted in two opposing graded effects, leading to differential outcomes based on the fragment’s origin. Specifically, MEK inhibition caused anterior blastemas to be more impaired in fragments derived from posterior regions (i.e., the original tail), while posterior blastemas were more impaired in fragments from anterior regions (i.e., the original head). Regeneration along the intact A-P axis following MEK inhibition exhibited the same A-P sensitivity trend, with more pronounced effects in the tail region of the animal.

### ERK activity is wound orientation and tissue position dependent

**Figure 3:**
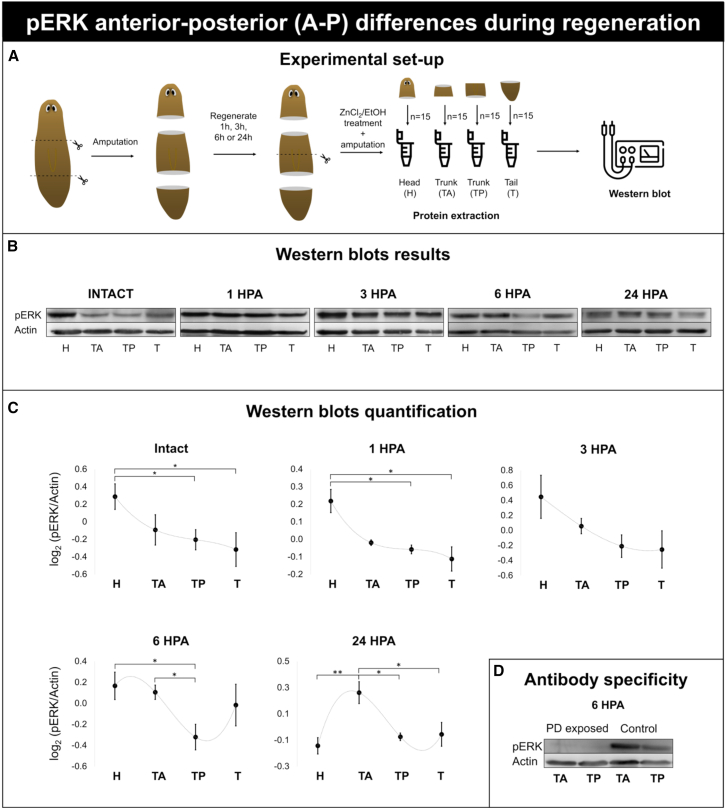
A-P differences in ERK activity during regeneration (A) Graphical representation of the experimental setup. The animals were transversely cut into three pieces and allowed to regenerate. At 1, 3, 6, or 24 HPA, the animals were treated with ZnCl_2_/EtOH. Trunk fragments were subdivided into anterior and posterior tissue regions and processed accordingly. Western blot analysis was performed on pooled tissue samples (*n* = 3) from head (H), trunk anterior (TA), trunk posterior (TP), and tail (T) fragments (*n* = 15). (B) Representative western blot results from one biological replicate at each time point (1, 3, 6, and 24 HPA). Lanes show the protein levels from H, TA, TP, and T fragments, left to right. Upper blots, pERK; lower blots, actin. pERK and actin were run on the same blot. (C) Quantified western blot results. pERK levels were log_2_ transformed and normalized to actin. Data represent the mean ± SEM from at least three independent experiments. The dashed gray line represents a polynomial fit included solely to guide the eye. Statistical significance between fragments of intact organisms and regenerating organisms was assessed using paired and unpaired Student’s *t* tests, respectively. ∗, *p* < 0.05; ∗∗, *p* < 0.01; ∗∗∗, *p* < 0.001. (D) Antibody specificity confirmed by western blot at 6 HPA in regenerating trunks with or without PD0325901 treatment.

**Figure 4:**
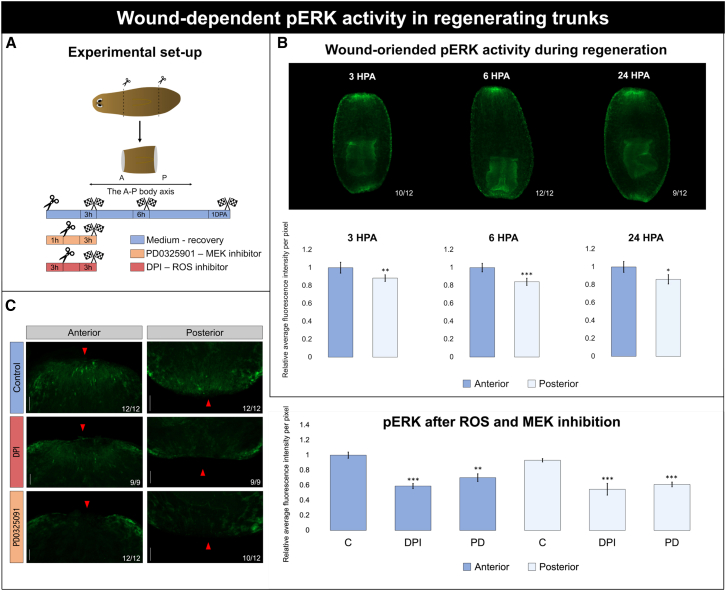
Wound-dependent pERK activity in regenerating trunks (A) Graphical representation of the experimental setup. The animals were transversally amputated anterior and posterior to the pharynx to generate a regenerating trunk with an anterior and posterior wound (*n* = 12). The animals used for pERK immunostainings were amputated and allowed to regenerate in medium (blue) after which they were fixated at 3, 6, and 24 HPA. To assess specificity of the pERK antibody, the animals were placed in either DPI (red) or PD0325901 (orange) to block ROS and MEK, respectively, for 1 h prior to amputation and up to 3 HPA. Next, the animals were fixed at 3 HPA. (B) Wound-oriented pERK activity during regeneration in trunk fragments. Representation of the wound-dependent pERK signal in regenerating trunk fragments is shown over time (3, 6, and 24 HPA) above the graph. The graph displays the quantified pERK levels at anterior (dark blue) and posterior (light blue) wounds, expressed as the relative average fluorescence intensity per pixel. Data represent the mean ± SEM from two independent experiments. Statistical significance was assessed using paired *t* tests. ∗, *p* < 0.05; ∗∗, *p* < 0.01; ∗∗∗, *p* < 0.001. (C) pERK after ROS and MEK inhibition. A visual representation of the effect of the pharmacological blockers PD0325901 and DPI on pERK levels is shown, with color code corresponding to the experimental setup in (A). Anterior wounds are shown on the left, with the wound facing up, and posterior wounds are shown on the right, with the wound facing down. The wound site is indicated with a red arrow. After DPI/PD treatment, the remaining green signal on the sides of the wound is autofluorescence, likely coming from secretory cells. The graph displays the quantified pERK levels at anterior (dark blue) and posterior (light blue) wounds after DPI or PD treatment, expressed as the relative average fluorescence intensity per pixel. Scale bars are 50 μm. Data represent the mean ± SEM from two independent experiments. Statistical significance was assessed using the Wilcoxon rank-sum test. ∗, *p* < 0.05; ∗∗, *p* < 0.01; ∗∗∗, *p* < 0.001.

Given that MEK inhibition affects regeneration depending on wound orientation and position (Figure 2), we analyzed ERK activity under homeostatic and regenerative conditions, using western blot analysis (Figure 3A). Antibody specificity was validated by PD treatment, which resulted in a clear decrease in pERK signal in both western blot and immunostaining analyses (Figures 3D and 4C).

### ERK activity is higher in anterior tissues of intact animals

In intact animals, the highest ERK activity was observed in the head, which progressively decreased toward the tail of the animal (Figures 3B and 3C). In particular, pERK levels in the heads (H) were significantly higher than those in the tails (T) and the posterior parts of the trunks (TP), with 1.5- and 1.4-fold differences, respectively.

### A-P differences in ERK activity are wound orientation dependent and re-established in regenerating trunks at 6 HPA

Following amputation, we assessed how the pERK gradient evolves in regenerating tissues at multiple time points (Figure 3). At 1 h post-amputation (HPA), the differences in ERK activity between the different fragments, though slightly less that the differences observed during homeostasis, were still present. pERK levels were significantly higher in head fragments than in posterior trunk and tail fragments (1.2- and 1.3-folds, respectively). No significant differences were found at 3 HPA, whereas after 6 HPA, A-P-specific differences in ERK activity became clearly established. Anterior trunk fragments showed significantly higher pERK levels than posterior trunk fragments (1.3-fold). At 24 HPA, anterior trunks maintained significantly elevated pERK levels compared with posterior trunks (1.2-fold). Moreover, pERK levels in heads severely decreased, resulting in significantly higher pERK levels in anterior trunk fragments compared with those in heads (1.3-fold). Comparison of fragments with their corresponding pre-amputation pERK levels showed that the pre-existing ERK activity changes following amputation (Figure S3).

The higher anterior-to-posterior pERK levels observed in the western blot analyses were confirmed by performing pERK immunostaining on non-exposed regenerating trunk fragments, which revealed consistently higher ERK activity in anterior wounds than in posterior wounds at 3, 6, and 24 HPAs (Figure 4B). Cells with well-defined projections displayed strong pERK signals at and around the wound site (Figure S4A). Within the epidermis, activated ERK was observed in both the cytoplasm and nucleus following amputation, consistent with its role as a transcriptional regulator (Figure S5).

In addition to local activation at the wound site, pERK-positive cells were also detected in tissues distant from the wound, including longitudinal muscle fibers, the epidermis, and fibrous structures in the intestinal tract (Figures S4B–S4D). After PD0325901 treatment during the earliest moments of regeneration, little to no pERK signal was detected at the wound sites, irrespective of its orientation (Figure 4C). A similar result was observed after diphenyleneiodonium chloride (DPI) treatment, which inhibits ROS, one of the upstream signals known to activate ERKs.

In summary, these findings collectively suggested that ERK activity is not only spatially graded in intact animals but also dynamically re-established during early regeneration on a whole-body scale in a time- and wound orientation-dependent manner.

### Wound-oriented redox dynamics align with ERK activity patterns

**Figure 5:**
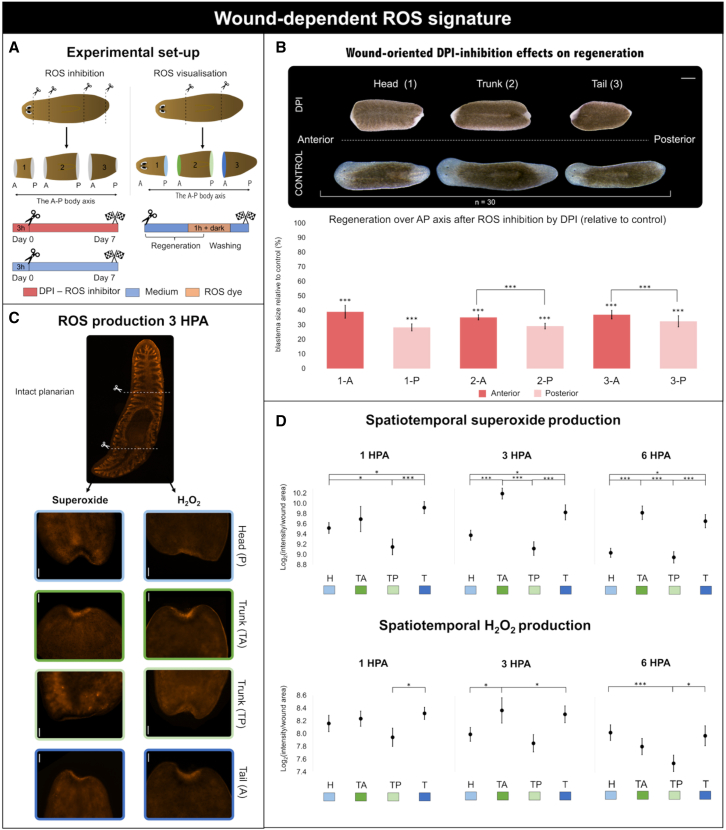
Wound-dependent ROS signature (A) Graphical representation of the experimental setup. For ROS inhibition, the animals were transversely amputated after removing the tips of the head and tail, which resulted in three fragments (1–3), each containing an anterior (A) and posterior (P) wound site (*n* = 30). ROS inhibition was carried out via DPI treatment (red), administered 3 h prior to amputation and continued for 7 days during regeneration. For ROS visualization, the animals were transversally cut anterior and posterior to the pharynx to generate a regenerating head (posterior wound), trunk (anterior and posterior wound), and tail (posterior wound) (*n* = 26). The animals were allowed to regenerate for 1, 3, or 6 HPA. Prior to real-time *in vivo* imaging, the animals were incubated in ROS dye for 1 h, after which they were washed and imaged. H_2_O_2_ production was visualized via peroxy orange 1 (PO1), and superoxide was visualized with dihydroethidium (DHE). (B) Wound-oriented DPI inhibition effects on regeneration at 7 DPA. Representation of regenerative outcomes after ROS inhibition at 7 DPA is shown above the graph. The graph displays quantified anterior (red) and posterior (light red) blastema sizes of all three fragments: fragment 1 (1-A and 1-P), fragment 2 (2-A and 2-P), and fragment 3 (3-A and 3-P), all relative to their corresponding control group (%). Data represent the mean ± SEM from at least three independent experiments. Statistical significance was assessed using one-way ANOVA. ∗, *p* < 0.05; ∗∗∗, *p* < 0.001. (C) Representative images of superoxide and H_2_O_2_ levels at 3 HPA at the wound site. In the upper part, superoxide in an intact animal is visualized; colors surrounding the image representing each wound site of heads (posterior wound, light blue), trunk (anterior wound, dark green), trunk (posterior wound, light green), and tail (anterior wound, dark blue) correspond to the colors of the graph showing the quantified levels. (D) Quantified spatiotemporal production of superoxide (upper graph) and H_2_O_2_ (lower graph). The intensity of the ROS dyes is expressed as the relative average fluorescence intensity per pixel. The graphs show the ROS levels for heads (H), trunk anterior (TA), trunk posterior (TP), and tails (T). Scale bars are 100 μm (C) and 500 μm (D). Data represent the mean ± SEM from at least three independent experiments. Statistical significance was assessed using one-way ANOVA. ∗, *p* < 0.05; ∗∗∗, *p* < 0.001.

Amputation-induced ROS have previously been shown to initiate MAPK-ERK signaling in planarians.^34^ To clarify how ROS dynamics relate to MAPK-ERK activation, we investigated the spatial redox differences in regenerating tissue fragments by blocking or visualizing ROS (Figure 5A).

Blocking ROS via DPI treatment significantly reduced blastema size at 7 DPA. This effect was A-P axis dependent as the posterior-oriented blastemas were smaller, relative to the control, than anterior-oriented blastemas (Figure 5B). This difference was observed regardless of the fragment’s original A-P position. To explore this orientation-dependent effect, we quantified superoxide and hydrogen peroxide (H_2_O_2_) levels at anterior-oriented wounds in trunks (TA) and tails (T), as well as at posterior-oriented wounds in trunks (TP) and heads (H) (Figures 5C and 5D). ROS dye specificity was confirmed by a significant reduction in fluorescence following DPI treatment and minimal to no background signal in unstained samples (Figure S6). Both superoxide and H_2_O_2_ levels peaked at 3 HPA and decreased by 6 HPA. Superoxide levels were consistently higher in anterior-oriented wounds across all time points, both within regenerating trunks and when comparing T and H (Figures 5C and 5D). TA produced significantly more superoxide than TP at 3 and 6 HPAs (2.1- and 1.8-folds, respectively). Similarly, T showed significantly higher levels than H and TP at all time points, with fold changes ranging from 1.3 to 1.71, and TA produced significantly more superoxide than H at 3 and 6 HPAs (1.76- and 1.72-folds, respectively). H_2_O_2_ levels followed a similar pattern, but the differences were more subtle. TA produced significantly more H_2_O_2_ than TP only at 3 HPA (1.43-fold). T had significantly higher levels than TP across all time points (1.30- to 1.37-fold), whereas no significant differences were observed between the TA and H fragments.

Together, these data indicated that early redox dynamics are governed primarily by wound orientation; superoxide is consistently produced more in anterior-oriented wounds, while H_2_O_2_ displays more transient and context-dependent differences that peak at 3 HPA.

### ERK signaling is potentially controlled by a redox-mediated feedback mechanism

**Figure 6:**
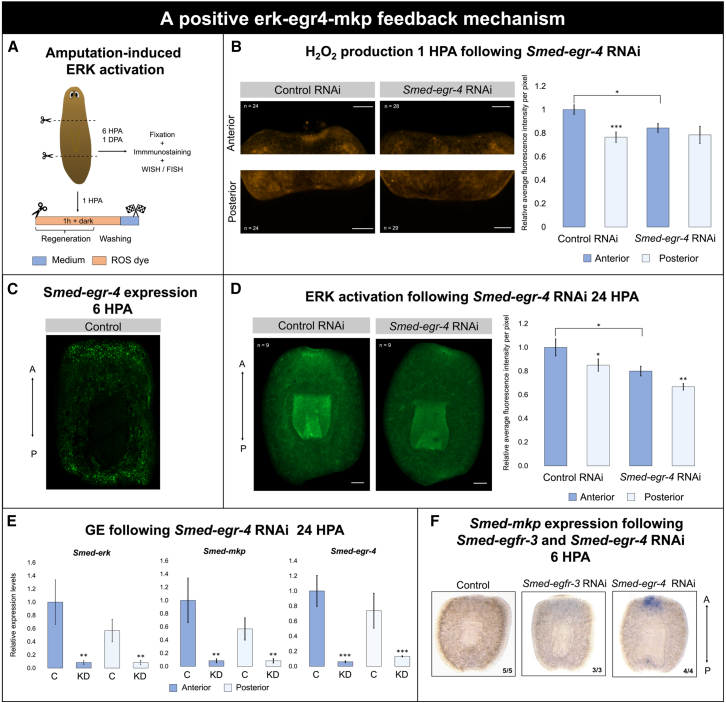
A positive ERK-EGR4-MKP feedback mechanism (A) Graphical representation of the experimental setup. Animals were transversally amputated anterior and posterior to the pharynx to generate a regenerating trunk (anterior and posterior wound). Following amputation, the animals were incubated in ROS dye (orange) for 1 H to visualize H_2_O_2_ at 1 HPA. Immunostaining, whole-mount *in situ* hybridization (WISH), and fluorescence *in situ* hybridization (FISH) were carried out by fixating animals at either 6 or 24 HPA. (B) Representative images of *in vivo* H_2_O_2_ production, using peroxy orange 1 (PO1) at 1 HPA in regenerating trunk pieces of control (*n* = 24) and *Smed-egr-4* RNAi-treated animals (*n* = 29). Data represent the mean ± SEM from two independent experiments. Statistical significance was assessed using Wilcoxon rank-sum test. ∗, *p* < 0.05; ∗∗, *p* < 0.01; ∗∗∗, *p* < 0.001. (C) Representative image of *Smed-egr-4* FISH in regenerating control trunks at 6 HPA (*n* = 3). (D) Immunohistochemical staining with anti-pERK in the anterior and posterior wounds of regenerating trunks of control and *Smed-egr-4* RNAi-treated animals at 24 HPA (*n* = 9). Data represent the mean ± SEM from one experiment. Statistical significance was assessed using Wilcoxon rank-sum test and Wilcoxon signed-rank test. ∗, *p* < 0.05; ∗∗, *p* < 0.01; ∗∗∗, *p* < 0.001. (E) Gene expression levels of *Smed-mkp*, *Smed-egr-4*, and *Smed-erk* after *Smed-egr-4* KD at 24 HPA. The animals were treated with *Smed-egr-4* RNAi (*n* = 50), after which anterior and posterior blastemas (*n* = 5 per condition) were pooled for each condition and snap-frozen for qPCR (*n* = 10 independent replicates). Color code: dark blue, anterior; light blue, posterior. Data represent the mean ± SEM from one experiment. Statistical significance was assessed using unpaired Student’s *t* tests. ∗, *p* < 0.05; ∗∗, *p* < 0.01; ∗∗∗, *p* < 0.001. (F) Relative changes in MAPK phosphatase (*Smed-mkp)* expression, shown via WISH in controls (*n* = 5) and planarians subjected to RNAi of *Smed-egr-4* (*n* = 4) and *Smed-egfr-3* (*n* = 3). The procedure was performed on trunk fragments at 6 HPA. All images are oriented with the anterior toward the top. Scale bars are 100 μm.

Previous research showed that ERK activation is regulated by *egfr-3* (epidermal growth factor receptor 3), while *egfr*-3 KD impaired ROS production after amputation.^34^ To further investigate the interplay between ROS and the MAPK-ERK signaling pathway, we analyzed how KD of *Smed-egfr-3* or *Smed-egr-4* affect antioxidant- and MAPK-related gene expression, pERK activity, and ROS production (Figures 6 and S7).

*Smed-egr-4* KD caused a significant downregulation of all AOX-related genes measured, independent of wound orientation (Figure S7B). Anterior H_2_O_2_ levels, on the other hand, were significantly reduced at 1 HPA, while posterior levels remained unaffected, thereby eliminating the natural A-P ROS asymmetry observed in controls (Figure 6B). The predominant expression of *Smed-egr-4* in the wound at 6 HPA further supports a role for *egr-4* in establishing early redox gradients (Figure 6C). Loss of *Smed-egr*-*4* also impaired ERK activation. ERK activity levels were reduced in both anterior- and posterior-oriented wounds at 24 HPA (Figure 6D), and *Smed-erk* transcript levels were significantly decreased at both wound sites (Figure 6E).

Early MAPK regulation was further found to be influenced by *egr-4*. *Smed-egfr-3* KD had no detectable effect on *Smed-mkp* (MAPK phosphatase) expression at 6 HPA, whereas *Smed-egr-4* KD caused a pronounced increase at this time point (Figure 6F). By 24 HPA, *Smed-mkp* expression was significantly reduced at both wound sites following *Smed-egr-4* KD, indicating a temporal shift in regulatory interactions (Figure 6E).

In conclusion, ROS levels are potentially regulated via an *egr-4*-mediated feedback loop, and *Smed-mkp* is possibly repressed by *Smed-egr-4* during the earliest moments of regeneration.

### Wound-oriented MAPK-ERK-Wnt/β-catenin interplay

**Figure 7:**
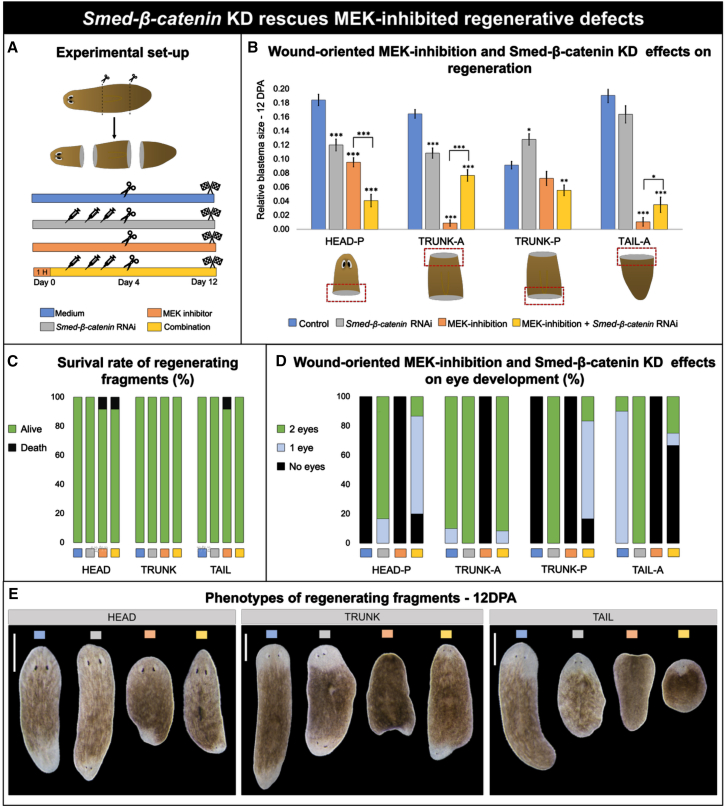
*Smed-β-catenin* KD rescues MEK inhibition-induced regenerative defects (A) Graphical representation of the experimental setup. Animals were transversally amputated (*n* = 14), generating three fragments (Head-P, Trunk-A or Trunk-P, and Tail-P). The animals were treated with culture medium (blue), *Smed-β-catenin* RNAi (gray), MEK inhibition (orange), or a combination of *Smed-β-catenin* RNAi and MEK inhibition (yellow). MEK inhibition was carried out via PD0325901 treatment, administered 1 h prior to RNAi and continued during and after the whole RNAi injection round. Regenerative outcomes were measured at 12 DPA, including relative blastema size and eye development. (B) Effects of wound-oriented MEK inhibition and *Smed-β-catenin* RNAi on regeneration. The graph displays quantified anterior and posterior blastema sizes after treatment with medium (blue), *Smed-β-catenin* RNAi (gray), MEK inhibition (orange), or a combination (yellow). Data represent the mean ± SEM from two independent experiments. Statistical significance was assessed using one-way ANOVA. ∗, *p* < 0.05; ∗∗, *p* < 0.01; ∗∗∗, *p* < 0.001. (C) Survival rate of regenerating fragments after the aforementioned treatments at 12 DPA. Color code: green, alive; black, death. (D) Eye development after 12 DPA of all three tissue fragments shown in (B). Color code: black, no eyes; blue, one eye; green, two eyes. (E) Representative phenotypes of heads, trunks, and tails at 12 DPA corresponding to the treatment outcomes shown in (B), (C), and (D).

**Figure 8:**
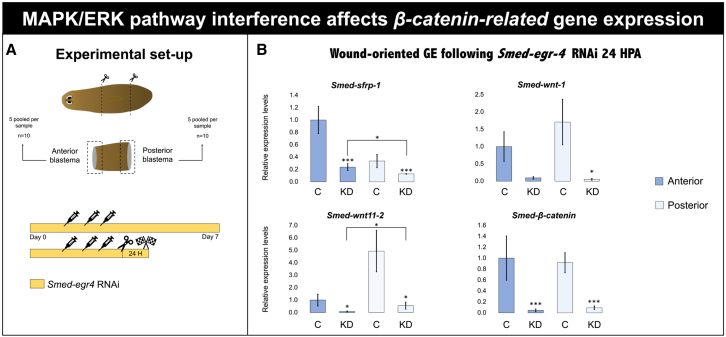
MAPK-ERK pathway interference affects β-catenin-related expression (A) Graphical representation of the experimental setup. The animals were transversally amputated above and below the pharynx, generating a regenerating trunk. The animals were then treated with *Smed-egr-4* RNAi (*n* = 50), after which anterior and posterior blastemas (*n* = 5 per condition) were pooled for each condition and snap-frozen for qPCR (*n* = 10 independent replicates). (B) Wound-oriented gene expression (GE) following *Smed-egr-4* RNAi at 24 HPA. The graph displays the anterior and posterior relative expression levels of *Smed-sFRP*, *Smed-Wnt1*, *Smed-Wnt11-2*, and *Smed-β-catenin.* Data represent the mean ± SEM from one experiment. Statistical significance was assessed using unpaired Student’s *t* tests. ∗, *p* < 0.05; ∗∗, *p* < 0.01; ∗∗∗, *p* < 0.001.

To explore whether the MAPK-ERK pathway specifies anterior regeneration by counteracting posterior-promoting pathways, we investigated its relation with *Smed-β-catenin* (Figures 7 and 8, and S8). Combining MEK inhibition via PD0325901 with *Smed-β-catenin* KD did not alter the overall survival rate, but it partially rescued the MEK inhibition-induced defects in anterior-oriented blastemas, leading to significantly larger blastema sizes in both trunk and tail fragments (Figures 7B and 7C). Correspondingly, while eyes failed to develop in anterior blastemas under MEK inhibition alone, eye formation was restored when MEK inhibition was combined with *Smed-β-catenin* KD (Figures 7D and 7E). In contrast, posterior regeneration in heads was significantly more reduced when MEK inhibition was combined with *Smed-β-catenin* KD compared to MEK inhibition alone. In posterior-oriented blastemas of trunks, no significant differences between these two treatments were observed. However, the presence of ectopic eyes in posterior blastemas was noted under the combined treatment (Figure 7E), consistent with the known phenotype of *Smed-β-catenin* KD. Underlying to these regenerative effects, the wound-dependent differences in pERK levels in regenerating trunks were no longer observed following *Smed-β-catenin* KD at 6 HPA (Figure S8).

Finally, to evaluate whether the pathways exert opposite regulatory effects, we examined the inverse relationship at the transcriptional level and found that *Smed-egr-4* KD led to a general downregulation of all β-catenin-associated genes at both wound sites (Figure 8B).

## Discussion

Body patterning is an evolutionarily conserved process across the animal kingdom.^39^^,^^40^ In organisms with remarkable regenerative capacities, such as planarians, patterning is essential for re-establishing the body axes and ensuring the spatial coordination of tissues and cells following injury.^3^ Recent work by Fan and colleagues demonstrated that injury-induced ERK activation propagates as an ultrafast wave, coordinating whole-body responses that are essential for successful regeneration.^26^ Anterior-posterior identity during regeneration is classically explained by opposing signaling gradients,^41^^,^^42^^,^^43^ with Wnt/β-catenin promoting posterior fate and activated ERK suggested to act as an anterior-specifying signal.^28^^,^^29^^,^^44^ Building on these insights, we aimed to further explore the mechanistic contributions and upstream signals that regulate early ERK activation during anterior regeneration.

### ERK activation in anterior regeneration

We found that ERK activation is locally and temporally regulated, depending on the A-P location of the wound, with its nuclear localization confirming a potential role in the regulation of cellular processes (Figure S5). Interfering with pERK activation, via blocking MEK activity, significantly affected both anterior and posterior regeneration in the regenerating trunks (Figure 2). Anterior regeneration was significantly more severely blocked than posterior regeneration, findings that align with the work of Owlarn et al. who showed that PD0325901 treatment inhibited regeneration in both anterior and posterior wound sites, with a more pronounced effect on anterior regeneration.^27^ Our study extends this conclusion by showing that within regenerating trunk fragments, ERK activation is wound orientation dependent. Higher pERK levels were produced in anterior-oriented wounds than in posterior-oriented wounds as early as 3–6 HPA, and this difference persisted at least until 24 HPA (Figures 3 and 4). The timing of A-P ERK differences precedes previously reported polarity decision windows in planarians, such as anterior notum (6–12 HPA) and posterior Wnt (18–48 HPA), indicating ERK as an early upstream regulator of polarity decisions.^18^^,^^35^^,^^45^^,^^46^ Notum is the only gene that is differentially expressed anteriorly between 6 and 12 HPA, and its early expression depends on ERK.^26^^,^^27^^,^^47^

The A-P difference in ERK activity is not restricted to regenerating tissues. In intact animals, we observed higher pERK levels in the anterior body than in the posterior (Figures 3B and 3C), suggesting that MAPK-ERK signaling reflects underlying positional information already present in uninjured animals and may contribute to guiding correct body re-establishment following injury.^28^^,^^29^^,^^48^ We validated these findings by showing that MEK inhibition had a more severe effect on anterior regeneration when the amputation site was more posterior, while the effect on posterior regeneration was milder (Figure 2B). In addition, regeneration was more affected in the tails when planarians were amputated longitudinally during MEK inhibition (Figure 2E). Differences in ERK activation along the original A-P axis (Figure 3C), inversely to the β-catenin differences, may suggest a potential (indirect) interplay between both pathways.^28^^,^^29^ Supporting this idea, *Smed-β-catenin* KD partially rescued MEK inhibition-induced anterior regeneration defects and eliminated the observed A-P differences in ERK activation (Figures 7 and S8). The inverse regulatory relationship was also observed as MAPK-ERK interference via *Smed-egr-4* KD clearly impaired Wnt/β-catenin signaling at the transcription level (Figure 8B).

Beyond local wound ERK activity, we observed ERK activity in tissues distant from the wound site (Figure S4). This observation supports recent findings by Fan et al., showing that ERK activation can propagate across tissues distant from the wound via muscle cells, acting as long-range signaling hubs upon wounding.^26^

### Redox-mediated MAPK-ERK activation

Previously, we demonstrated that ROS initiate regeneration by activating the MAPK-ERK pathway.^34^^,^^38^ In this study, we show that ROS production levels in trunks exhibit a A-P-related pattern similar to ERK activity after amputation (Figures 4B, 5C, and 5D). We observed significantly higher levels of both superoxide and H_2_O_2_ in anterior-oriented wounds, peaking at 3–6 HPA (Figures 5C and 5D), while in posterior-oriented wounds, a less pronounced ROS burst was observed. Opposing wound orientations produce distinct ROS levels, whereas fragments with identical wound orientations show similar ROS dynamics (Figures 5C and 5D), indicating that A-P differences in ROS are determined by wound orientation, rather than by the fragment’s original A-P identity, and, thus, represent a wound-induced positional signal rather than a pre-existing pattern, as seen for ERK.

Pharmacological inhibition of ROS with DPI severely affected posterior regeneration, whereas MEK inhibition more strongly impaired anterior regeneration (Figures 2B, 2E, and 5B). The higher sensitivity of posterior regeneration to ROS inhibition may reflect a broader role of ROS beyond ERK activation, as ROS are known to modulate other relevant signaling pathways, including Wnt/β-catenin, which is essential for posterior identity in planarians.^49^ In addition, the use of DPI, a broad flavoprotein inhibitor, may contribute to this effect by simultaneously targeting multiple ROS-generating enzymes and redox-dependent processes beyond ERK regulation.^50^ Although the complexity in ROS targets may initially appear counterintuitive in the context of the redox-mediated ERK activation initiating anterior regeneration, it is consistent with previous findings by Pirotte and colleagues, who showed that DPI treatment disrupted patterning and led to ectopic expression of the well-characterized anterior markers such as *Smed-notum*, *Smed-th*, and *Smed-cintillo,* which were observed in posterior blastemas.^38^ Together, these findings imply that while ROS are essential for wound-dependent ERK activation, their signaling roles likely influence multiple pathways whose interactions during regeneration remain to be elucidated.

### A feedback mechanism between ERK, EGR-4, and MKP to regulate the activation state of the MAPK-ERK signaling pathway

ROS, ERK, Egr-4, and MKP co-occur at the wound site shortly after amputation,^22^^,^^27^^,^^29^^,^^38^^,^^51^ with higher anterior levels of ROS and pERK at 6 HPA, and an increased anterior expression of *Smed*-*egr-4* at later time points.^22^ In addition to *egr-4* acting downstream of the MAPK pathway, our results indicated that *Smed-egr-4* KD strongly reduced H_2_O_2_ production at the wound sites (Figure 6B), decreased pERK activity at 24 HPA, and increased *Smed-mkp* expression at the anterior wound site of regenerating trunks at 6 HPA (Figures 6D and 6F). These changes in *Smed-mkp* expression suggest the potential involvement of *Smed-egr-4* in regulating A-P-related redox signaling by inhibiting MKP activity, thereby preventing pERK dephosphorylation and inactivation. The inactivation of pERK via MKP is well described and has been demonstrated in *D. japonica*, where pERK levels increased following *DjmkpA* KD.^51^^,^^52^^,^^53^

These findings correspond to those of our previous study, which showed decreased ROS production after KD of *Smed-egfr-3*, which acts upstream of *Smed-egr-4*.^22^^,^^34^ In contrast, KD of *Smed-egfr-3* did not lead to an upregulation of *Smed-mkp* in anterior blastemas at 6 HPA (Figure 6F). Fraguas et al. showed that *Smed-egr-4* is expressed in two phases: an early *Smed-egfr-3*-independent phase up until 24 HPA, and a late *Smed-egfr-3*-dependent phase after 24 HPA, which could explain this difference.^22^ After 24 HPA, *Smed-egr-4* KD reversely affected *Smed-mkp* expression relative to that at 6 HPA, resulting in a downregulation of *Smed-mkp* at both anterior and posterior wounds (Figure 6E). This agrees with earlier observations in *D. japonica* showing that treatment with U0126, an MEK inhibitor, reduced *Djmkp* expression at anterior wound sites at 24 HPA.^51^

Here, we demonstrate the importance of *Smed-egr-4* in regulating ERK activity. The hypothesized feedback loop is illustrated in Figure S9. On the basis of our proposed feedback mechanism and the wave-like propagation of ERK activation observed after amputation by Fan and colleagues,^26^ we hypothesize that ERK activity in planarians might propagate through a relay mechanism, rather than through passive diffusion in a gradient manner, similar to the one proposed in *D. melanogaster*, *C. elegans*, and other model systems.^54^^,^^55^^,^^56^^,^^57^^,^^58^ Though further investigation is needed, this relay mechanism may involve sequential ERK activation via the positive egr4-ROS feedback loop (Figure S9).

### Limitations of the study

This study dissects the interplay between redox dynamics and MAPK-ERK signaling during early planarian regeneration, but several limitations should be considered. Pharmacological inhibition and RNA interference (RNAi) were used to perturb pathway activity. Pharmacological inhibitors such as DPI and PD have broad effects, as DPI inhibits multiple flavoprotein-dependent redox enzymes, and PD broadly suppresses MAPK signaling, limiting pathway specificity and preventing discrimination between individual ROS sources or redox processes. To address these limitations, we complemented pharmacological inhibition with targeted KD of individual pathway components. Moreover, KD efficiency was assessed using multiple independent readouts, including ROS measurements, pERK levels, and transcriptional analyses of redox- and MAPK-responsive genes. *In vivo* ROS measurements relied on fluorescent probes that enable spatial and temporal comparisons but provide relative, not absolute, quantification and may be influenced by probe kinetics and tissue properties. To strengthen these analyses, multiple ROS-sensitive dyes were employed. For western blot analyses, pERK levels were normalized to β-actin rather than total ERK, as commercially available total ERK antibodies did not yield a reliable signal in our hands, which may limit direct assessment of changes in ERK phosphorylation relative to total ERK abundance. In addition, ERK activation and related effects were predominantly assessed at the tissue level. As a result, potential heterogeneity among individual cell types or stem cell states could not be resolved in this study. Future single-cell or spatially resolved analyses will be required to directly link ERK activity dynamics to specific cell identities and fate decisions during regeneration.

## Resource availability

### Lead contact

Requests for further information and resources should be directed to and will be fulfilled by the lead contact, Martijn Heleven (martijn.heleven@uhasselt.be).

### Materials availability

The study did not generate new unique reagents.

### Data and code availability

• Data reported in this paper will be shared by the lead contact upon request.
• No new code was generated in this study.
• Any additional information required to reanalyze the data reported in this paper is available from the lead contact upon request.

## Acknowledgments

The authors thank Natascha Steffanie and Ria Vanderspikken for their skillful technical assistance and Silke Wilms, Lucia Malikova, Joke Aerts, and Merel Hufkens for their contributions during their internships. This work was supported by the Research Foundation Flanders (FWO, project G047921N), the Ministerio de Ciencia, Innovación y Universidades (project PID2021-126958NB-I00), and the Agència de Gestió d’Ajuts Universitaris i de Recerca de la Generalitat de Catalunya (project 2021SGR00293). The research presented in this publication was carried out with infrastructure funded by the European Marine Biological Resource Centre (EMBRC) Belgium- FWO international research infrastructure (I001621N). Microscopy was made possible by the Research Foundation Flanders (FWO, project I001222N).

## Author contributions

Conceptualization, M.H., F.C., and K.S.; methodology, M.H., M.D.M., V.J., and K.B.; software, M.H.; validation, M.H. and M.D.M.; formal analysis, M.H.; investigation, M.H., M.D.M., and V.J.; writing – original draft, M.H. and K.S.; writing – review & editing, M.H., M.D.M., V.J., K.B., F.C., and K.S.; visualization, M.H.; resources, F.C. and K.S.; supervision, F.C. and K.S.; funding acquisition, F.C. and K.S.; project administration, K.S.

## Declaration of interests

The authors declare no conflict of interest.

## STAR★Methods

### Key resources table

**Table:** 

REAGENT or RESOURCE	SOURCE	IDENTIFIER
**Antibodies**		

Phospho-p44/42 MAPK (Erk1/2) (Thr202/Tyr204) (D13.14.4E) XP® Rabbit mAb	Cell Signaling Technology	*Cat#4370S; RRID:**AB_2315112*
Anti-beta Actin antibody - Loading Control	Abcam	Cat# ab8227, RRID: AB_2305186
m-IgGκ BP-HRP Antibody	Santa Cruz Biotechnology	Cat# sc-516102; RRID: AB_2687626
Goat Anti-IgG (H&L) Polyclonal HRP Antibody	AgriSera	Cat# AS09 602; RRID: AB_1966902
Mouse Anti-Synapsin Monoclonal Antibody (anti-SYNORF1), Unconjugated	Developmental studies hybridoma Bank	Cat# 3C11; RRID: AB_528479
Mouse Anti-Muscle Fibers Antibody (6G10-2C7), Unconjugated	Developmental studies hybridoma Bank	Cat# 6G10-2C7; RRID: AB_2619613
Goat anti-Mouse IgG (H+L) Cross-Adsorbed Secondary Antibody, Alexa Fluor™ 568 Conjugated	Molecular Probes	Cat# A-11004; RRID: AB_2534072
Goat anti-Rabbit IgG (H+L) Cross-Adsorbed Secondary Antibody, Alexa Fluor™ 488 Conjugated	Molecular Probes	Cat# A-11008; RRID: AB_143165

**Chemicals, peptides, and recombinant proteins**		

Diphenyleneiodonium chloride	Enzo Life Sciences	Cat#BML-CN240-0010
PD0325901	Sigma-Aldrich (Roche)	Cat#PZ0162
cOmplete™, EDTA-free Protease Inhibitor Cocktail	Sigma-Aldrich (Roche)	Cat#1187358000
PhosSTOP™	Sigma-Aldrich (Roche)	Cat#4906845001
Peroxy Orange 1 (PO1)	Sigma-Aldrich	Cat#SML0688

**Critical commercial assays**		

ROS-ID® Superoxide detection kit	Enzo Life Sciences	Cat#ENZ-51012
Western Lightning Plus, Chemiluminescent Substrate	pErkinElmer	Cat#NEL103E001EA

**Experimental models: Organisms/strains**		

*Schmidtea mediterranea*	This paper	N/A

**Oligonucleotides**		

Primers for RNAi probes, see Table S1	This paper	N/A
Primers for qPCR, see Table S2	This paper	N/A

**Software and algorithms**		

Fiji (ImageJ)	Fiji	RRID:SCR_002285
R Project for Statistical Computing	R Development Core Team	RRID:SCR_001905
RSudio	Team RStudio	RRID:SCR_000432
Nikon NIS Elements software	Nikon	RRID:SCR_014329
ZEISS ZEN Microscopy Software	ZEISS	RRID:SCR_013672

### Experimental model and study participant details

For all experiments, an asexual strain of the freshwater planarian *Schmidtea mediterranea* was used. The animals were originally obtained from the University of Barcelona and have been maintained in the Smeets lab for more than 10 years. Age was not controlled as an experimental variable and was not considered a meaningful parameter under our experimental conditions. Only fully regenerated animals were used to initiate new experiments. No ethical approval was required, as S. mediterranea is an invertebrate species.

*Schmidtea mediterranea* was continuously kept in the dark at a constant temperature of 20°C and maintained in culture medium composed of ultrapure H_2_O containing 1.6 mM NaCl, 1 mM CaCl_2_, 1 mM MgSO_4_, 0.1 mM MgCl_2_, 0.1 mM KCl, and 1.2 mM NaHCO_3_. Once a week, animals were fed with veal liver. All animals were starved for at least 7 days prior to experiments. Amputation was performed to artificially induce regeneration. The specific amputation strategy employed depended on the research questions being addressed, which is explained in the respective sections and figures.

### Method details

#### Pharmacological interference

##### Inhibition of reactive oxygen production

Diphenyleneiodonium chloride (DPI, Enzo Life Sciences), a non-specific flavoprotein inhibitor was used to interfere with several electron transporters, and subsequently block ROS production. Planarians were exposed to 3 μM DPI for 3 h prior to- and up to seven days post amputation (7 DPA). Exposure medium was refreshed every other day. Because of the hydrophobic character of DPI, the final exposure solution contained 0.01 % (v/v) dimethyl sulfoxide (DMSO, Sigma Aldrich), which was shown to not affect the planarian regeneration process.^59^ To study a possible ROS gradient along the anterior-posterior (A-P) axis, the tip of the head (i.e. region before the eyes) and tip of the tail were eliminated followed by amputating the animal in three pieces. This resulted in a head, trunk, and tail pieces that all contained an anterior and posterior oriented wound site. After treatment with the ROS inhibitor, animals were gently washed and placed into fresh medium prior to phenotypic screening.

##### Inhibition of MEK activity

To prevent the phosphorylation and activation of ERK, the activity of MEK was blocked with the chemical compound PD0325901 (Sigma-Aldrich). PD0325901 was dissolved in DMSO (Sigma Aldrich) and used at a concentration of 10 μM. Animals were pre-exposed to PD0325901 for 1h prior to amputation and for either 5, 7, or 14 DPA. Exposure solutions were changed every other day.

To study the potential existence of an ERK gradient along the A-P axis, two distinct amputation setups were used. The first setup was designed to assess whether A-P ERK activation plays a role during regeneration in a context that requires repatterning of positional information, potentially influenced by the spatial A-P gradients prior to amputation (e.g., β-catenin). In this setup, the tip of the head (i.e., the region anterior to the eyes) and the tip of the tail were removed before cutting the animal into three pieces. This resulted in the formation of a regenerating head, trunk, and tail piece, each containing both an anterior and posterior-oriented wound site. The second setup allows to investigate regenerative effects of MEK inhibition within the context of the existing A-P gradients (e.g., β-catenin). Therefore, animals were cut along the sagittal plane, i.e. between the eyes, to create a wound along the A-P axis. The midpoint of the length of the animal was then determined, and head and tail regions were each divided into three equal parts. The midpoint of the animal’s total body length was determined and used as a reference point to divide the animal into three equal regions: anterior, middle, and posterior.

#### RNA interference

Gene knockdown (KD) via RNA interference (RNAi) was performed with double stranded RNA (dsRNA) probes of *Smed-β-catenin*, *Smed-egfr-3*, *Smed-mek*, *Smed-erk*, and *Smed-egr-4.* dsRNA probes were generated by an *in vitro* transcription system (T7 RibomaxTM Express RNAi System, Promega) as previously described.^60^ Primer sequences are summarized in Table S2. Probe injections were performed on three consecutive days on intact animals for two weeks, leading to a total of 6 injections. Each time, animals were injected with 32.2 nL of 1500 ng/μl dsRNA probe. Injections were executed ventrally in the anterior part of the planarian, between the pharynx and photoreceptors, using the Nanoject II (Drummond Scientific, Broomall, PA, USA). The control group was injected with the same volume of ultrapure H_2_O and followed the same injection schedule. The day following the last injection, animals were transversally cut.

#### Real-time *in vivo* ROS detection

The compounds Peroxy Orange 1 (PO1, Sigma Aldrich–SML0688), and Dihydroethidium (DHE, ROS-ID superoxide detection kit, Enzo–51012) were used to specifically stain intracellular hydrogen peroxide (H_2_O_2_) and superoxide. First, the specificity of the ROS dyes was confirmed (Figure S5). Raw fluorescence intensity distributions showed that DPI exposure caused a reduction in peak intensity frequency together with a leftward shift, indicating both fewer ROS-positive pixels and lower overall signal intensity (Figure S5B). Unstained ROIs exhibited only a single low-intensity peak (10–16), confirming that all detectable fluorescence in stained samples originates from the dyes. Quantifying total pixel intensities normalized to the wound area further supported these observations, control-stained samples displayed significantly higher hydrogen peroxide and superoxide levels than DPI-treated or unstained samples (Figure S6C).

To investigate the dynamics of ROS production at the amputation site, the *in vivo* detection of ROS was conducted at various time points, i.e. 1, 3, and 6 HPA. Planarians were cut in 3 parts to generate a regenerating head, trunk, and tail to study A-P dependent ROS production. Prior to imaging, tissue fragments were incubated for 1h in 20 μM PO1 (for H_2_O_2_) or 0.5 μM DHE (for superoxide). The ROS assay was also applied to *Smed-egr-4* RNAi animals and their corresponding controls. One day after completing the second RNAi cycle, animals were transversally amputated to generate regenerating trunks. These fragments were incubated for 1 h in 20 μM PO1 and imaged at 1 HPA. After staining, fragments were gently rinsed with fresh culture medium, and immobilized in 2.5 % (w/v) low melting point (LMP) agarose (Invitrogen, 16520-050) as previously described.^61^ Samples were imaged using the widefield (WF) mode of a Zeiss LSM900 confocal microscope (Carl Zeiss Microscopy GmbH). Imaging settings were kept constant across all samples within each experiment.

#### Western blotting

To study pERK levels during regeneration, western blot (WB) was performed as described by Fan and colleagues.^26^ Animals were transversally cut above and below the pharynx, to generate three pieces that were allowed to regenerate in cultivation medium for 1, 3, 6 or 24 HPA. Next, the fragments were incubated in 100 nM ZnCl_2_ diluted in ethanol, for 45 min at 4°C, to stop ongoing ROS-mediated signaling events prior to applying additional cuts. Trunks were cut in two pieces generating an anterior and posterior trunk fragment without altering ERK activation via amputation-induced ROS. In total, fifteen tissue fragments per group (i.e. head, trunk anterior, trunk posterior, tail) for each timepoint were sampled and three biological replicates were prepared for each group. After fixation, protein extracts were prepared by homogenizing snap-frozen tissue fragments in lysis buffer (4.8 M urea, 1.6 % SDS, 104 mM DDT) containing protease and phosphatase inhibitors (cOmplete protease Inhibitor Cocktail and PhosSTOP, ROCHE). The homogenization process involved using a tissuelyser for mechanical homogenization combined with multiple freeze-thaw cycles. Extracts were divided in a pellet and supernatant by centrifugation (max speed, 2 min, 4 °C). The total protein content of the supernatant was quantified using the NanoDrop ND-1000 (NanoDrop Technologies). After quantification, extracts were diluted in 1X loading buffer (LB) (4X LB: 40 mM NaPi, pH 7.0; 40 % glycerol; 10 % SDS; 2 mg/ml DTT; 0.67 mg/ml bromophenol blue).

Approximately 80 mg of protein was loaded on a discontinuous 10 % polyacrylamide SDS-page gel. Proteins were transferred on a nitrocellulose membrane using the Mini-Protean cassette system (Bio-rad) at 100 V for 1h15 min at 4 °C. The membrane was briefly washed with TBSTx (20 mM Tris-HCl, pH 7.5, 150 mM NaCl, 0.05 % Tween-20) and blocked in 5% non-fat Milk in TBSTx for 1h at room temperature. Next, the membrane was probed with the primary pERK antibody (1:1000, Cell signaling 4370) followed by an HRP-conjugated secondary antibody (goat-anti-rabbit, Agrisera,1:10000 diluted). Washes were carried out with TBSTx and the protein bands were visualized using Western Lightning Plus ECL substrate (pErkinElmer). After detection, the membrane was incubated in stripping solution (7% acetic acid, 5% MetOH, diluted in ultrapure H_2_O) for 15 min at room temperature with gentle shaking. It was then re-blocked and re-probed with a β-Actin antibody (1:5000, Abcam ab8227), and processed as described above. Specificity of the primary pERK antibody was validated by PD0325901 treatment, which resulted in strongly reduced or absent band intensity (Figure 3D).

#### Immunohistochemistry

Several immunostainings were carried out: (i) to visualize developmental effects on the central nervous system (CNS) after PD0325901 treatment, (ii) to characterize the spatiotemporal localization of pERK after amputation, and (iii) to co-localize pERK in muscle cells. The following antibodies were used: (i) anti-SYNORF-1, a monoclonal antibody specific for synapsin, used as a pan-neural marker for the CNS (Developmental Studies Hybridoma Bank, 1:10 dilution), (ii) anti-Phospho-p44/42 MAPK (Erk1/2) monoclonal antibody that specifically detects endogenous levels of p44 and p42 MAP Kinase when dually phosphorylated at Thr202 and Tyr204 of ERK1, or singly phosphorylated at Thr202 (Cell Signaling Technology, 1:250 dilution), (iii) 6G10-2C7, a muscle specific monoclonal antibody (Developmental Studies Hybridoma Bank, 1:50 dilution). Immunostaining of the CNS, muscle, and pERK were carried out as described before.^33^^,^^62^ Briefly, animals were euthanized and mucus was removed by immersion in 2 % HCl for 5 minutes on ice. Next, tissues were fixed in 4 % formaldehyde diluted in 1X PBSTx (10 × PBS: 1.37 M NaCl, 27 mM KCl, 100 mM Na_2_HPO_4_, 20 mM KH_2_PO_4_ in ultrapure H_2_O), 0.3 % (v/v) Triton X-100) for 15 minutes at room temperature (RT), followed by overnight bleaching in 6 % H_2_O_2_ in PBSTx under a cold light source (3000K). Subsequently, the fragments were blocked in blocking solution (1 % (w/v) BSA in PBSTx) for 4 hours at RT, and incubated overnight at 4 °C in the primary antibody for 18 hours. Animals were rinsed with PBSTx for 6-8 hours, followed by a 1-hour incubation in blocking solution at RT. Finally, animals were incubated with the appropriate Alexa-conjugated antibody for 3 hours at RT, washed with PBSTx for at least 2 hours, and mounted in ImmuMount (Thermo Fisher Scientific). For pERK immunostaining, phosphatase inhibitors (PhosSTOP, Roche) were added to each solution following mucus removal, except for the bleaching solution. Specificity of the primary pERK antibody was validated by PD0325901 and DPI treatment, which resulted in a strongly reduced antibody signal (Figure 4C). Additional no-primary-antibody controls further confirmed antibody specificity (Figure S10).

#### Whole mount *in situ* hybridization

The expression of MAPK phosphatase (MKP) was assessed using colorimetric *in situ* hybridization with NBT/BCIP (Roche) chemistry, following the protocol previously described.^63^ Probes were synthesized using the DIG RNA (SP6/T7) Labeling Kit (Roche), starting from a purified PCR product of the gene of interest generated with gene-specific primers. The forward primer (5’-3’): GACAATTTACGTTGTCCAACA and reverse primer (5’-3’): GTCCGGCGCCGTTTGACCCA, were used for the development of the DIG-labeled *Smed-mkp* probe. After mounting, samples were analyzed, and bright-field images were captured using a MZ16F stereomicroscope (Leica) equipped with a ProgRes C3 camera (Jenoptik).

#### Fluorescent-intensity measurements

After the detection of *in vivo* superoxide or H_2_O_2_ production in amputated planarians using Dihydroethidium (DHE, ROS-ID superoxide detection kit, Enzo–51012) and Peroxy Orange 1 (PO1, Sigma-Aldrich, SML0688), we quantified the fluorescent signal derived from the probes. Due to the highly autofluorescent nature of planarian tissues, autofluorescent signals within the same excitation/emission range as the probes were also measured and accounted for. For all experimental conditions (1-, 3-, and 6 HPA) at least 10 samples for each tissue fragment were imaged, and three independent experiments were performed. Fluorescent images were captured in widefield (WF) mode using a ZEISS LSM900 confocal microscope (Carl Zeiss Microscopy GmbH), maintaining constant fluorescent lamp intensity across tissue fragments within each experiment. To quantify the signal intensity within the regions of interest (i.e. the wound site), the raw data of the images were analyzed using the ZEISS ZEN blue software version 3.7.7 (Carl Zeiss Microscopy GmbH). First, the wound region of interest (ROI) was defined based on the presence of white, unpigmented tissue. The product of the signal intensity value and the number of pixels corresponding to that intensity for each pixel in the ROI was calculated. This was done for all intensities present within the ROI. The total signal intensity was then calculated by taking the sum of these products. To normalize for the size of the wound, the total signal intensity was divided by the area of the ROI to obtain an average signal intensity per unit area. To ensure that autofluorescence did not contribute to the anterior-posterior (A/P) differences observed in regenerating tissue fragments, controls without dye labeling were included at 1 HPA. These control samples were treated identically and imaged under the same conditions as dye-incubated samples. All controls displayed a low and stable autofluorescence signal, which did not change depending on the position of the tissue fragment along the A/P axis or the orientation of the wound (Figure S5). Additionally, the specificity of these ROS dyes has been previously described in detail by our lab through various approaches.^34^^,^^38^^,^^61^^,^^64^ Fluorescent intensity measurements for pERK immunostainings were conducted using the same methodology (Figures 4B, 4C, S7, and S10).

#### Microscopy

Samples were imaged with a ZEISS LSM900 microscope equipped with an Axio Observer 7, a 120V HXP illuminator, and GaASP-PMT or multialkali PMT detectors. The following objectives were used: Objective EC Plan-Neofluar 10x, 0.3 NA; Objective Plan-Apochchromat 20x, 0.8 NA; Objective LD C-Apochromat 40xW, 1.1 NA; Objective Plan-Apochromat 63xoil, 1.40 NA. Fluorophores were excited with the following lasers: Diode laser 405 nm (5 mW), Diode laser 488 nm (10 mW), Diode laser 561 nm (10 mW). All images used for quantification analysis were acquired with 16-bit depth and for confocal imaging a pinhole of 1.00 AU was used in all experiments. Specifically, to visualize pERK nuclear translocation a 3.36 μm thick z-stack, consisting of 25 slices was taken with the airyscan super resolution mode. Afterwards, this image stack was used to create a colocalization map of nuclear pERK (section 3.3.3).

#### RT-qPCR

To study the A-P differences in expression of MAPK/ERK targets after treatment with RNAi, 5 anterior and 5 posterior blastemas were isolated and pooled from regenerating trunks at 24 HPA. Primer sequences of the genes of interest are listed in Table S2. At least ten pooled samples were prepared for each treatment. RNA extraction and subsequently quantitative real-time PCR (qPCR) was carried out as previously described.^65^ Snap-frozen samples were first dissolved in 100 μL of lysis buffer (Qiagen) containing 1 % β-mercaptoethanol. RNA extraction was performed following a standard phenol:chloroform protocol, with RNA precipitation achieved using sodium acetate and ethanol. RNA concentration and purity were assessed using the Nanodrop ND-1000 spectrophotometer. Genomic DNA was removed using the Turbo DNA-free kit (Invitrogen). Next, cDNA was synthesized using the Superscript III first-strand synthesis supermix (Thermo Fisher Scientific). The resulting cDNA samples were diluted (1/8) and measured using the QuantStudio 5 (Thermo Fisher Scientific). Stable reference genes were determined using the geNorm algorithm in qbase^+^ (Biogazelle)^66^ (Figure S11). Knockdown efficiency was assessed using the same qPCR approach on intact animals prior to amputation, with four pooled samples per treatment, consisting of 10 animals (Figure S1B).

#### Phenotypic screening

To assess the effects of RNAi- and pharmacological treatments, the effect on the newly formed tissue was studied. Development of the photoreceptors was also taken into account and its degree of success was scored as two eyes, one eye, no eye, or two faint eyes. Pictures were taken on 4, 7 or 14 DPA with a binocular microscope and a Nikon SMZ600 camera using Nikon NIS Elements software. Blastema sizes were measured with ImageJ (v1,49p, National Institute of Health) and expressed relative to the total body. In experiments assessing the effects of ROS and MEK inhibitors on A–P–dependent blastema formation, each tissue fragment was normalised to its corresponding control fragment to take into account pre-existing A-P differences in blastema size related to the fragment’s original position along the A–P body axis (Figure S12).

### Quantification and statistical analysis

All statistical analyses were performed using the R studio statistical software version 2024.04.2+764. Normality was checked with Shapiro-Wilk. For independent groups, an unpaired Student’s t-test was performed or a one-way ANOVA followed by a Tukey post-hoc correction for multiple testing. For dependent groups, paired t-tests were conducted to compare paired observations. P- values < 0.05 were considered as statistically significant. When assumptions of normality were not met after data transformation (Square root or Log transformation), a Kruskal-Wallis Rank Sum test and a Wilcoxon Rank Sum test were performed. In this case Holm-correction was used to adjust for multiple testing. For statistical analysis and visualization, ROS fluorescence intensities and Western blot quantifications of ERK activity were log_2_-transformed. For clarity, fold differences are described in the main text, based on non-transformed group means.
