# Supplementary material for: Redox-modulated ERK dynamics support wound-dependent tissue formation during early planarian regeneration
Source: iScience. 2026 Apr 8;29(5):115640. doi: 10.1016/j.isci.2026.115640 (PMC13185785; doi:10.1016/j.isci.2026.115640)
Supplement: Document S1. Figures S1–S12 and Tables S1–S3 [file mmc1.pdf]

iScience, Volume *29*

Supplemental information

Redox-modulated ERK dynamics support wound-dependent tissue formation during early planarian regeneration

Martijn Heleven, Maria Dolores Molina, Vincent Jaenen, Karolien Bijnens, Francesc Cebrià, and Karen Smeets

Supplemental figures

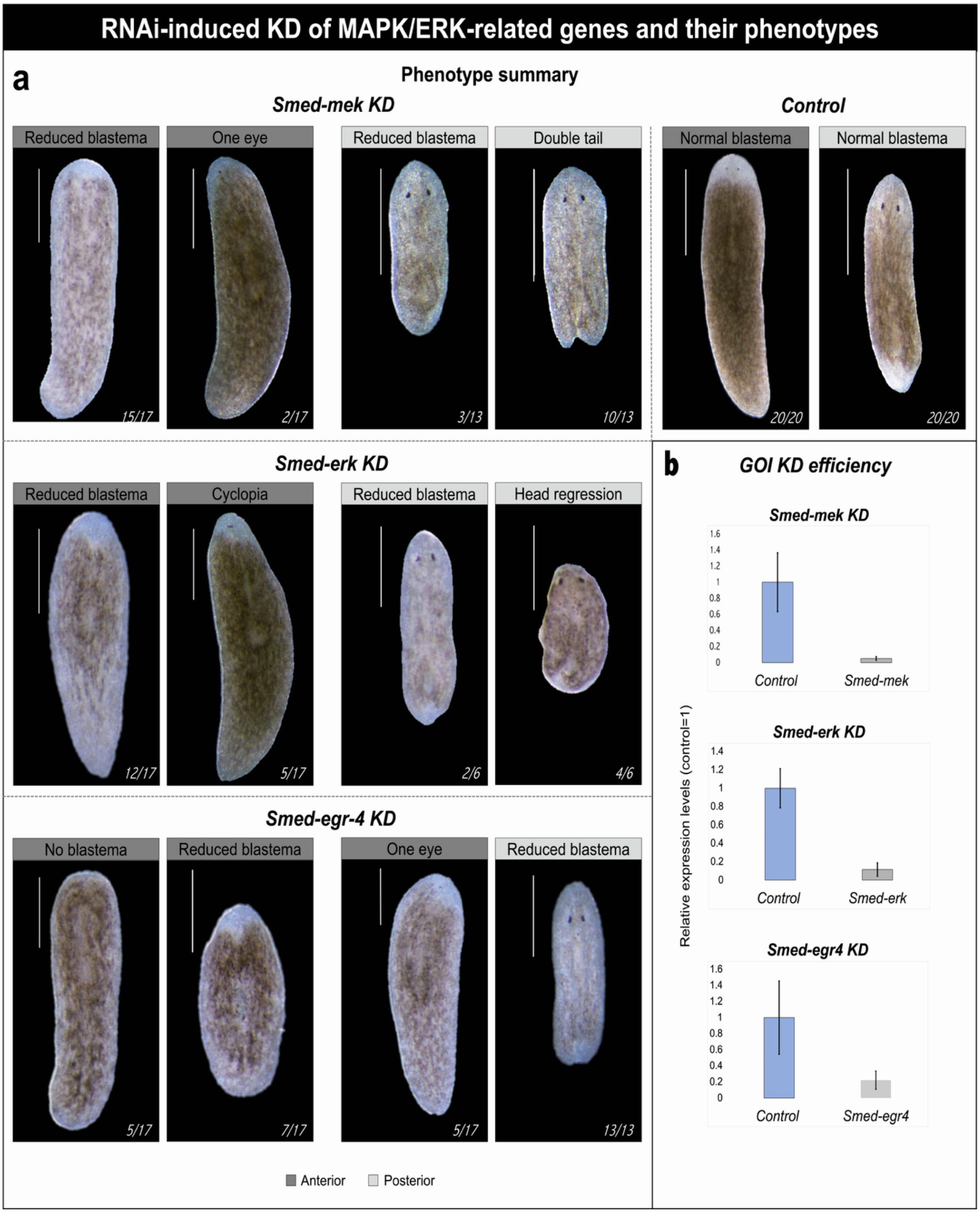

Supplementary figure 1: RNAi-induced KD of MAPK/ERK-related genes and their phenotypes. a) Representative images showing the range of regeneration phenotypes observed following RNAi-mediated knockdown of *Smed-mek*, *Smed-erk*, and *Smed-egr-4* (Fig. 1). The number of animals displaying each phenotype is indicated (n/total). Dead animals were excluded in the total number, but are summarized separately in Fig. 1B. **b)** Knockdown efficiency of each gene of interest, shown as relative expression levels compared to control animals (normalized to 1). Color code phenotypes: dark grey= regenerating tail (anterior blastema), light grey= regenerating head (posterior blastema). Scale bars are 1000µm.

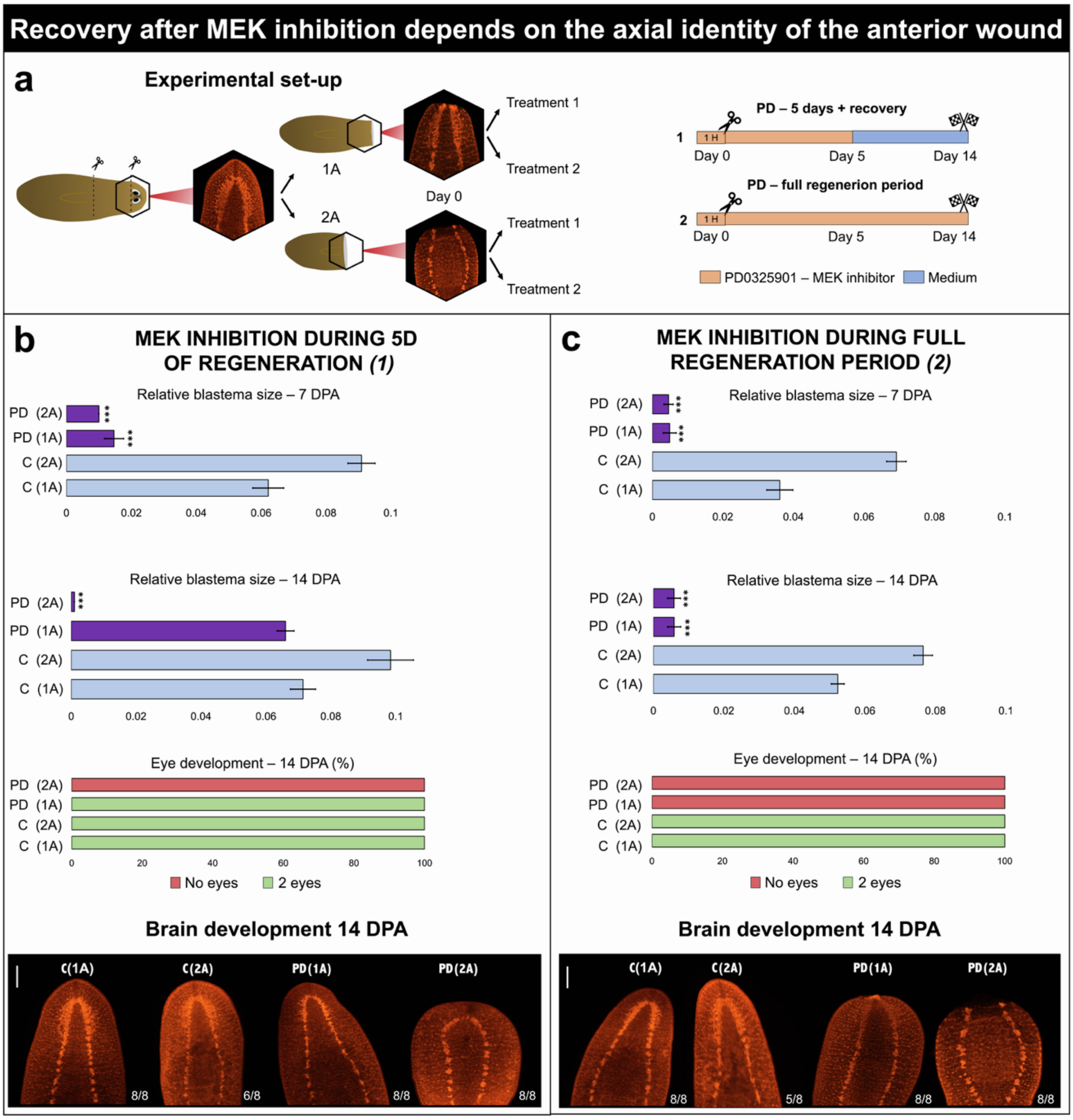

Supplementary figure 2: Recovery after MEK inhibition depends on the axial identity of the anterior wound. a) Graphical representation of the experimental setup: on the left, the amputation setup is displayed. The central nervous system (CNS) is visualized by anti-synapsin immunostaining in an intact planarian before amputation and after amputation just posterior to the eyes (fragment 1A) or just anterior to the pharynx (fragment 2A). The tissue fragments (1A and 2A) received two different treatments (1 and 2) (n=10). Corresponding treatment conditions are presented on the right side of the panel, with control animals not exposed. MEK-inhibited animals were treated with the MEK inhibitor, PD0325901 (orange), in two ways: (1) planarians were exposed 1 hour before amputation and during the first 5 days post-amputation (DPA), followed by a recovery period in medium (blue) until 14 DPA; (2) planarians were exposed 1 hour before amputation and throughout the entire 14-day regeneration period. **b-c)** Effects of MEK inhibition on regeneration after treatment 1 are shown in panel b, and the effects after treatment 2 are shown in panel c. From top to bottom: The graphs show differences in blastema sizes relative to the full body size (µm²) of PD-treated (purple) animals, either fragment 1A or fragment 2A at 7 DPA and 14 DPA. Eye (%) and brain regeneration at 14DPA are shown after both treatments. Color code "relative blastema size": purple = PD0325901, light blue = controls; Color code "eye regeneration": red = no eyes, green = 2 eyes. Scale bars: 200 µm. Statistical significance was assessed using the Student's *t* test (*: $p < 0.05$), (**: $p < 0.01$), (***: $p < 0.001$).

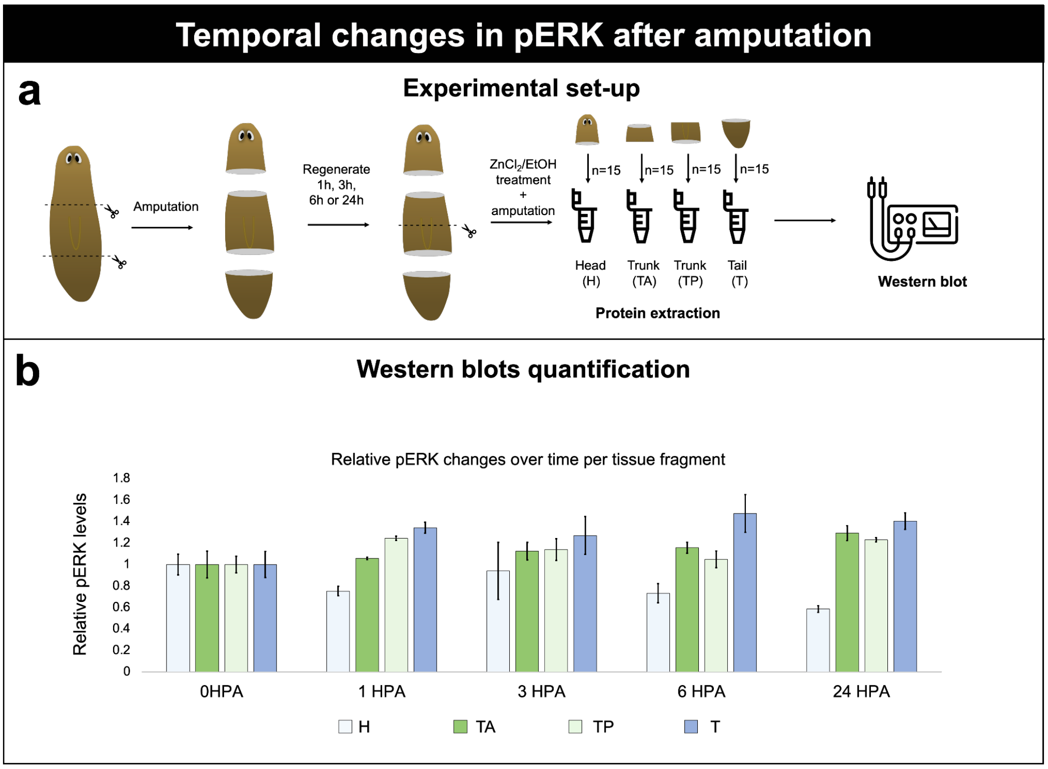

Supplementary figure 3: Temporal changes in pERK after amputation. a) Graphical representation of the experimental setup: animals were transversely cut into three pieces and allowed to regenerate. At 1-, 3-, 6-, or 24-hours post-amputation (HPA), animals were treated with ZnCl2/EtOH. Trunk fragments were subdivided into anterior and posterior tissue regions and processed accordingly. Western blot analysis was performed on pooled tissue samples (n=3) from head (H), trunk anterior (TA), trunk posterior (TP), and tail (T) fragments (n = 15). **b)** Quantified Western blot results. pERK levels were normalized to actin, and expressed relative to the corresponding fragment prior to amputation (0HPA). Data represent mean ± SEM from at least three independent experiments.

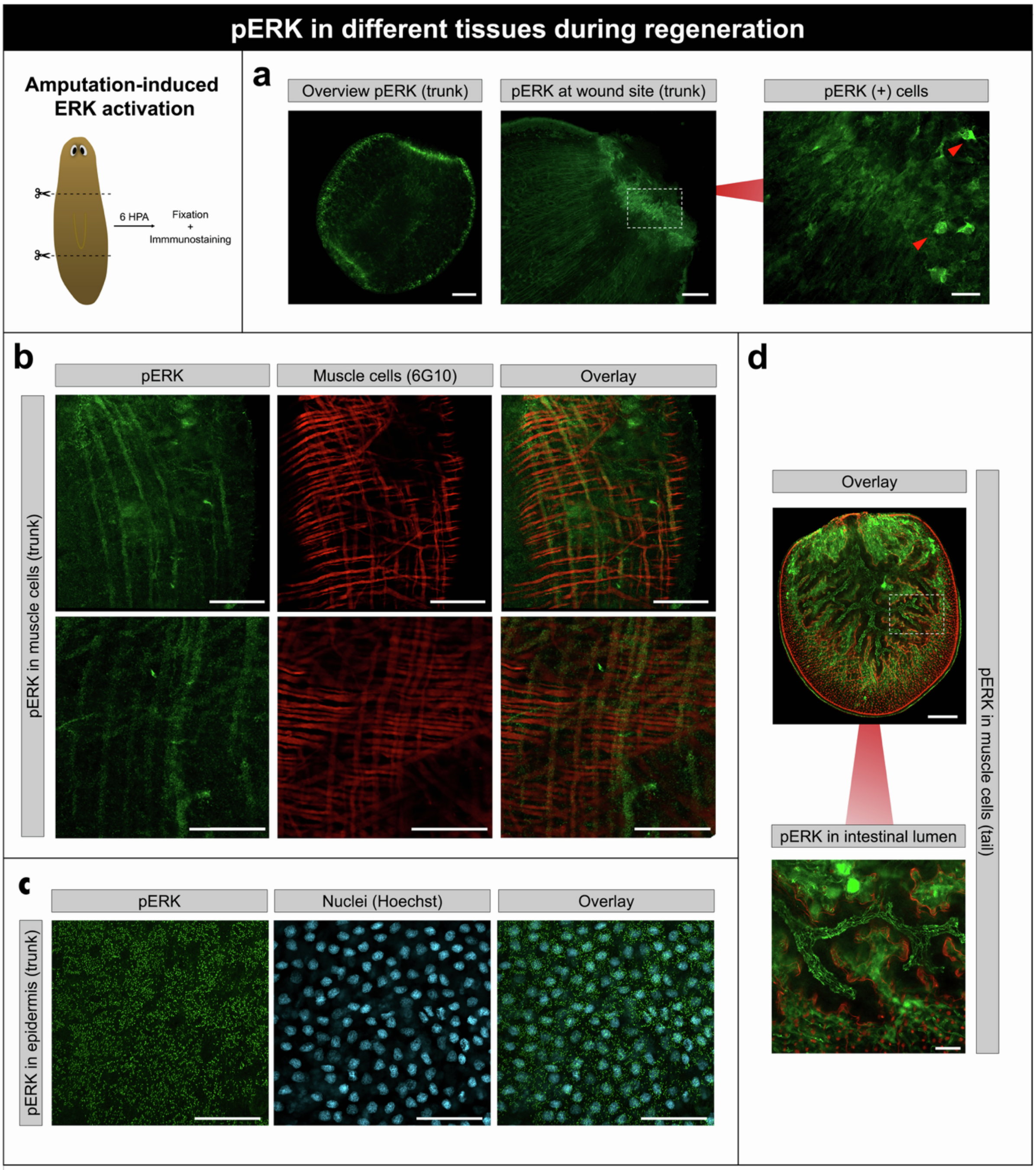

Supplementary figure 4: pERK in different tissues during regeneration. Top left: Schematic representation of amputation-induced ERK activation. Amputation above and below the pharynx generated regenerating trunks, while amputation only below the pharynx generated regenerating tails. **a)** pERK activation at the wound site. From left to right: an overview of ERK activation at both wound sites in a regenerating trunk (Scale bar = 200µm), a focused image showing pERK at the wound site (Scale bar = 50µm), with pERK-positive cells showing clear projections indicated by red arrowheads (Scale bar = 20µm). **b)** Localization of pERK in longitudinal muscle cells close to the wound site in regenerating trunks (Scale bar = 50µm). This section combines pERK (green) staining with 6G10 muscle staining (red). The overlay of both stainings is shown alongside the individual staining patterns. **c)** pERK presence in the epidermis in regenerating trunks (Scale bar = 50µm). pERK (green) staining is combined with nuclei staining (Hoechst). The overlay of both stainings is shown alongside the individual staining patterns. **d)** Overview showing pERK localization in filamentous-like structures within the intestinal lumen of a regenerating tail (Scale bar = 100µm), as observed by the zoomed in image combining pERK (green) and 6G10 (red) immunostaining (Scale bar = 20µm). All images are displayed with the anterior side up.

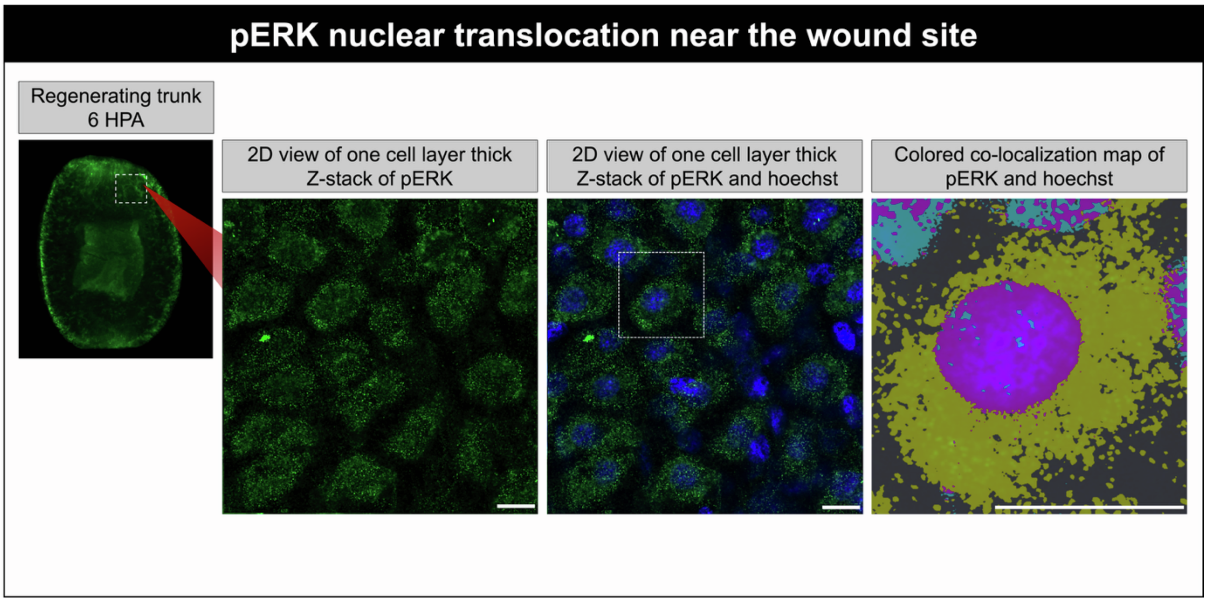

Supplementary figure 5: Epidermal pERK nuclear translocation near the wound site. From left to right: (i) pERK present at both wound sites of a regenerating trunk at 6 HPA, (ii) 2D maximum intensity projection view of a one cell layer thick Z-stack showing pERK (green), (iii) 2D maximum intensity projection view of a one cell layer thick Z-stack showing both pERK (green) and nuclei (blue), and (iiii) Colored co-localization map of pERK and Hoechst. Color code: green/yellow= pERK, blue= Hoechst, purple=co-localization. Scale bar= 5 μm.

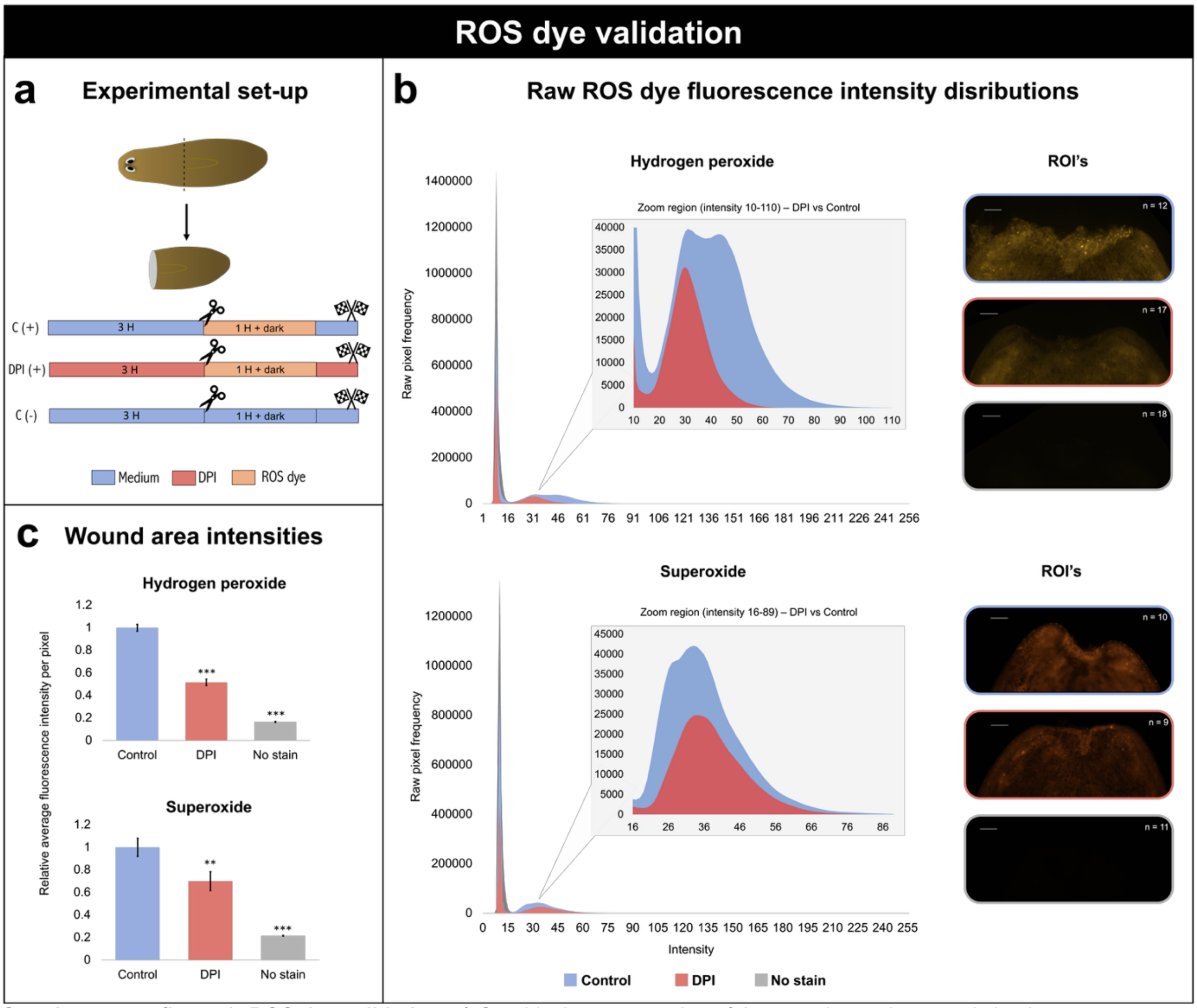

Supplementary figure 6: ROS dye validation. **a)** Graphical representation of the experimental set up. Animals were amputated prior to the pharynx to generate regenerating tails with an anterior wound. Prior to amputation animals were incubated either in medium, or DPI for 3 hours. After amputation, animals were incubated with the ROS dyes for 1h in the dark. Negative controls were also placed in the dark but without ROS dye. **b)** Raw ROS dye fluorescence intensity distributions. Top: Hydrogen peroxide intensity distribution of the ROI's on the left. A zoomed cut off is displayed (intensity 10-110) to highlight the differences between control and DPI treated samples. Top right: representative images of H_2O_2 staining at anterior wounds 1 HPA of control (n=12), DPI treated (n=17), and no stained animals (n=18). Bottom: Superoxide intensity distribution of the ROI's on the left. A zoomed cut off is displayed (intensity 16-89) to highlight the differences between control and DPI treated samples. Bottom right: representative images of superoxide staining at anterior wounds 1 HPA of control (n=10), DPI treated (n=9), and no stained animals (n=11). **c)** Wound area intensities of the ROS dyes. Graph represents the total pixel intensity present within the wound area in control, DPI treated, or no stained animals. Color code panel: blue= control, red= DPI treated, grey= no stain. All panels are oriented with the anterior towards the top. Scale bars are 100 µm. Data represent mean ± SEM from two independent experiments. Statistical significance was assessed using the unpaired Student's *t* test (*: $p < 0.05$), (**: $p < 0.01$), (***: $p < 0.001$).

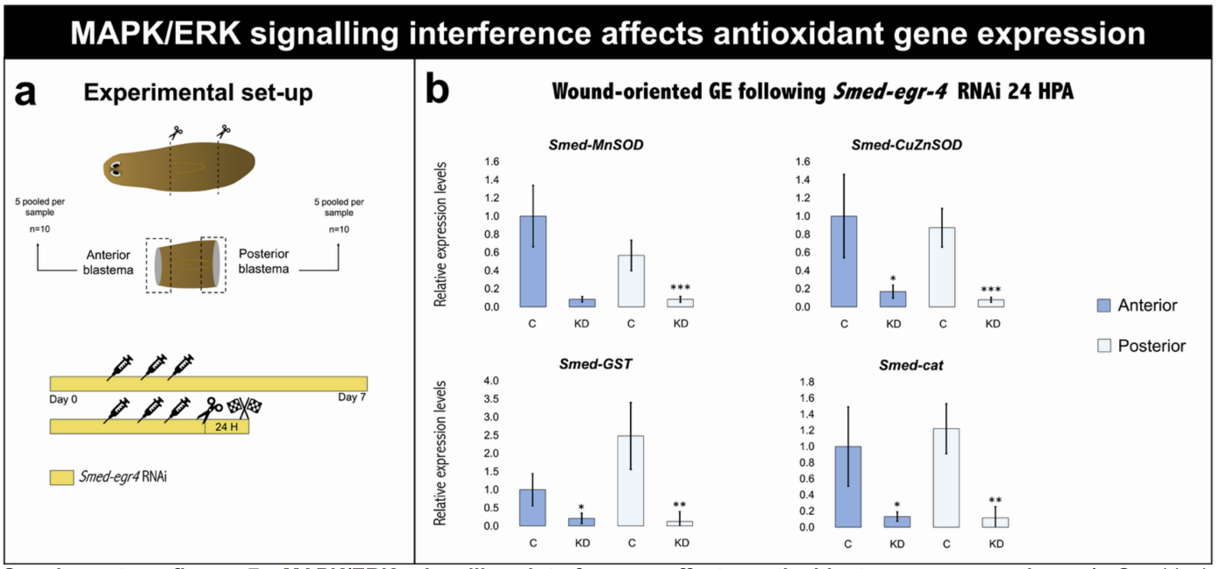

Supplementary figure 7: MAPK/ERK signalling interference affects antioxidant gene expression. a) Graphical representation of the experimental setup: animals were transversally amputated anterior and posterior to the pharynx to generate a regenerating trunk (anterior and posterior wound). Prior to amputation, animals were treated with a two week injection period targeting *Smed-egr-4*. After amputation, animals were allowed to regenerate for up to 24 HPA after which anterior and posterior blastemas were pooled per 5 to generate 10 biological samples for each treatment condition. **b)** Gene expression levels of *Smed-CuZnSOD*, *Smed-MnSOD*, *Smed-Catalase*, and *Smed-GST* following *Smed-egr-4* RNAi-mediated KD at 24 HPA. Color code: dark blue= anterior, light blue= posterior. Data represent mean ± SEM from one experiment. Statistical significance was assessed using the unpaired Student's *t* test (*: $p < 0.05$), (**: $p < 0.01$), (***: $p < 0.001$).

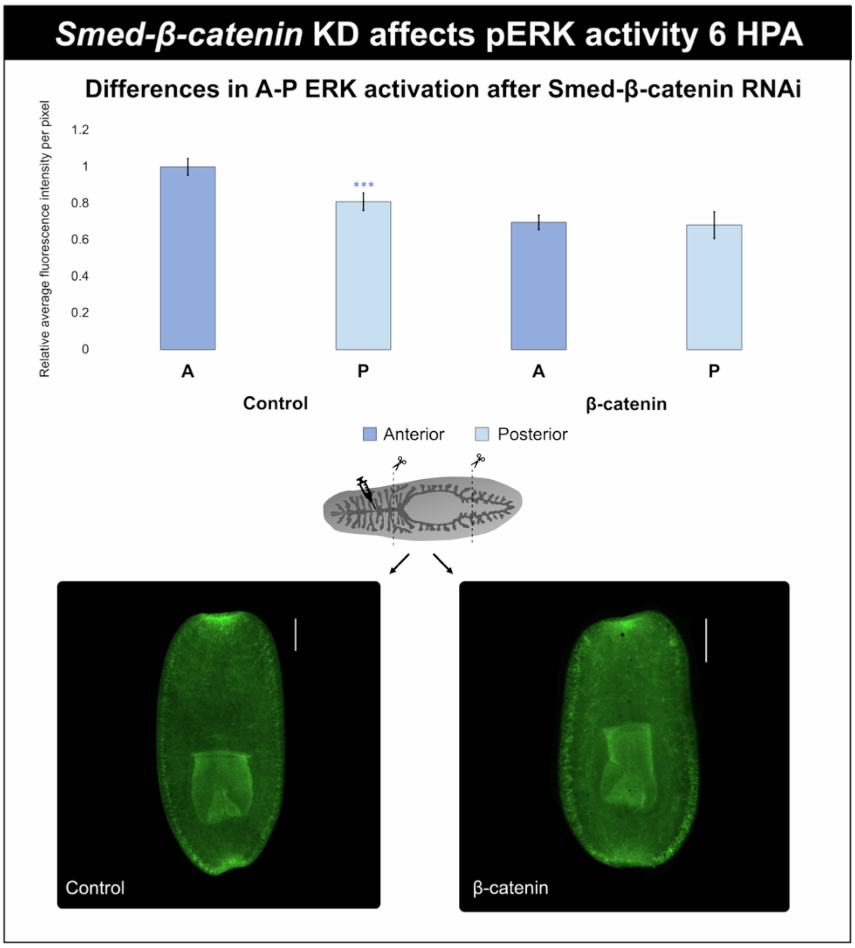

Supplementary figure 8: ***Smed-β-catenin* KD affects pERK activity 6 HPA.** The effects of *Smed-β-catenin* RNAi-mediated KD on differences in A-P pERK levels are represented. After injection, animals were transversally amputated anterior and posterior to the pharynx to generate a regenerating trunk with an anterior and posterior wound (n=12). After 6 HPA, animals were fixed for a pERK immunostaining. A representation of the wound-dependent pERK signal in regenerating trunk fragments in control and RNAi-treated animals is shown below the graph. The graph displays the quantified pERK levels at anterior (dark blue) and posterior (light blue) wounds and is expressed as the relative average fluorescence intensity per pixel. Scale bars = 200 µm. Data represent mean ± SEM from two independent experiments. Statistical significance was assessed using the paired Student's *t* test (*: $p < 0.05$), (**: $p < 0.01$), (***: $p < 0.001$).

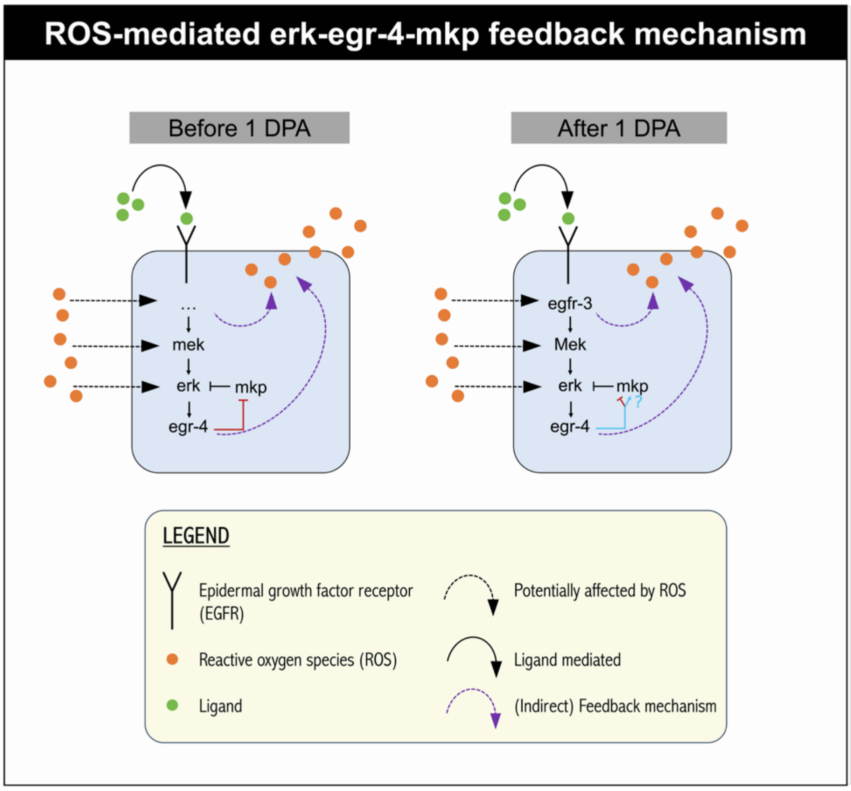

Supplementary figure 9: Proposed ROS-mediated erk-egr-4-mkp feedback mechanism during regeneration. The MAPK/ERK signaling cascade resulting in downstream activation of the Smed-egr-4 transcription factor is shown at two time points: before 1 DPA (on the left) and after 1 DPA (on the right). At both timepoints, Smed-egfr-3 and Smed-egr-4 influence ROS levels. Before 1 DPA, egr-4 is regulated in an egfr-3-independent manner and can inhibit Mitogen-Activated Protein Kinase Phosphatase (MKP) activity to maintain ERK phosphorylation levels. After 1 DPA, egr-4 is regulated in an egfr-3-dependent manner and does not inhibit MKP activity. However, the exact mechanism in which egr-4 influences MKP activity after 1 DPA remains to be elucidated. In both graphical representations the mode of action of reactive oxygen species (ROS) is indicated. ROS can activate the pathway by acting on the ligand, the ligand-receptor interaction, or redox-sensitive cysteine residues on either the receptor or downstream kinases. Color code “dots”: orange= reactive oxygen species, green= ligands. Color code “arrows”: red inhibition line: activity inhibition, black straight-line arrows= activation via phosphorylation, black dotted line arrows= Potential ROS-mediated activation, purple dotted line arrows= (indirect) feedback mechanism, blue straight arrow= unknown effect.

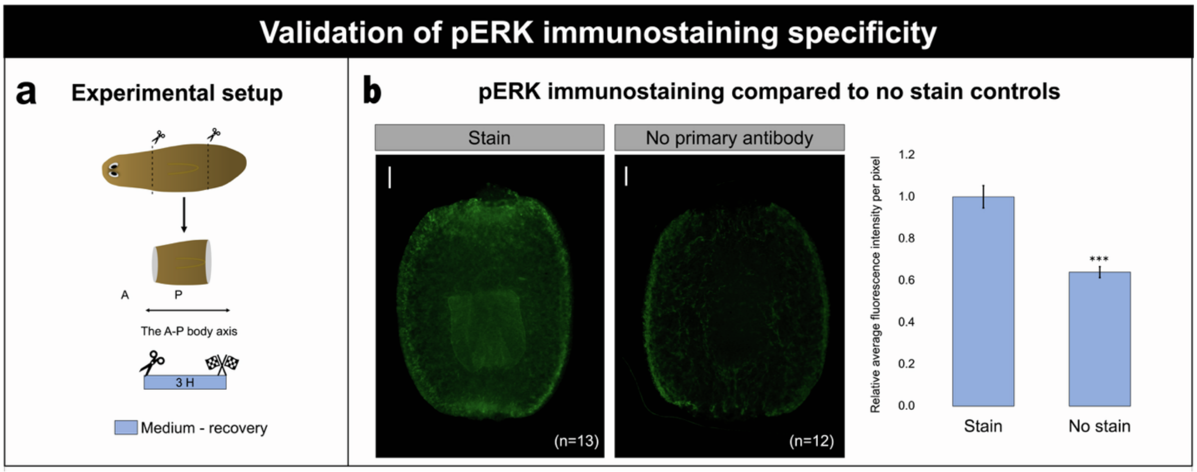

Supplementary figure 10: Validation of pERK immunostaining specificity. a) Graphical representation of the experimental setup. Animals were transversely amputated anterior and posterior to the pharynx to generate a regenerating trunk fragment with an anterior and a posterior wound site. After amputation, animals were allowed to regenerate for up to 3 HPA, after which they were fixed and processed for pERK immunostaining. **b)** pERK immunostaining in stained controls compared with no primary antibody controls. Left: representative images showing wound-dependent pERK signal in regenerating trunk fragments in stained control and no-stain animals. Right: quantification of whole-body pERK levels, expressed as the relative average fluorescence intensity per pixel. Scale bars = 100 µm. Data represent mean ± SEM from one experiment. Statistical significance was assessed using the paired Student's *t* test (*: $p < 0.05$), (**: $p < 0.01$), (***: $p < 0.001$).

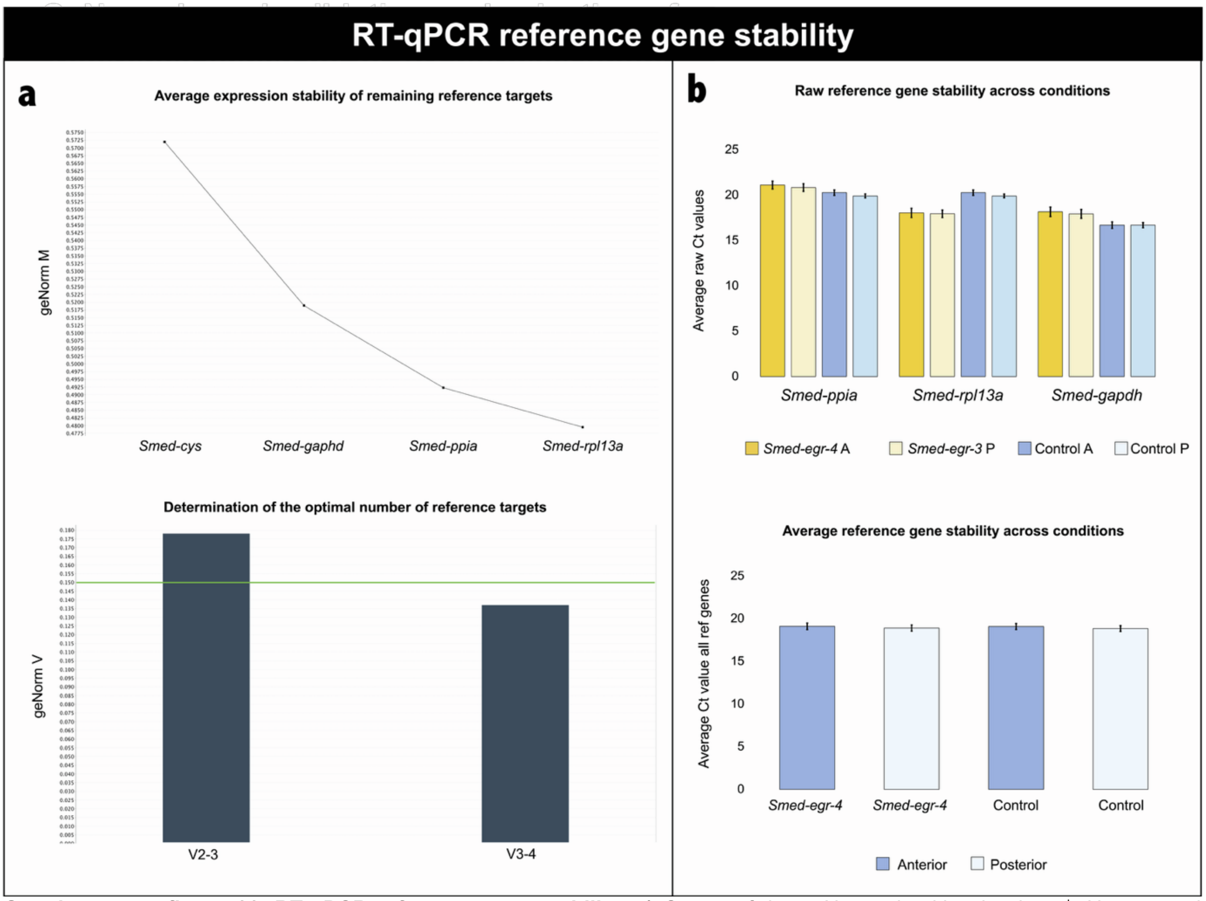

Supplementary figure 11: RT-qPCR reference gene stability. a) Output of the geNorm algorithm in qbase+. Upper graph shows the average expression stability of remaining reference targets expressed as geNorm M. Lower graph shows the optimal number of reference targets (3) expressed as geNorm V (*Smed-gapdh*, *Smed-ppia* and *Smed-rpl13a)*. **b)** Overview of differences in raw Ct values across experimental conditions. The upper graph shows raw Ct value stability of the reference genes across conditions and wound orientations. Color code: yellow = *Smed-egr-4* A, light yellow = *Smed-egr-4* P, blue = Control A, light blue = Control P. The lower graph shows the average reference Ct value stability across conditions and wound orientations, calculated as the mean Ct of the three reference genes shown in the upper graph. Color code: blue = anterior, light blue = posterior. Data represent mean ± SEM from three independent experiments.

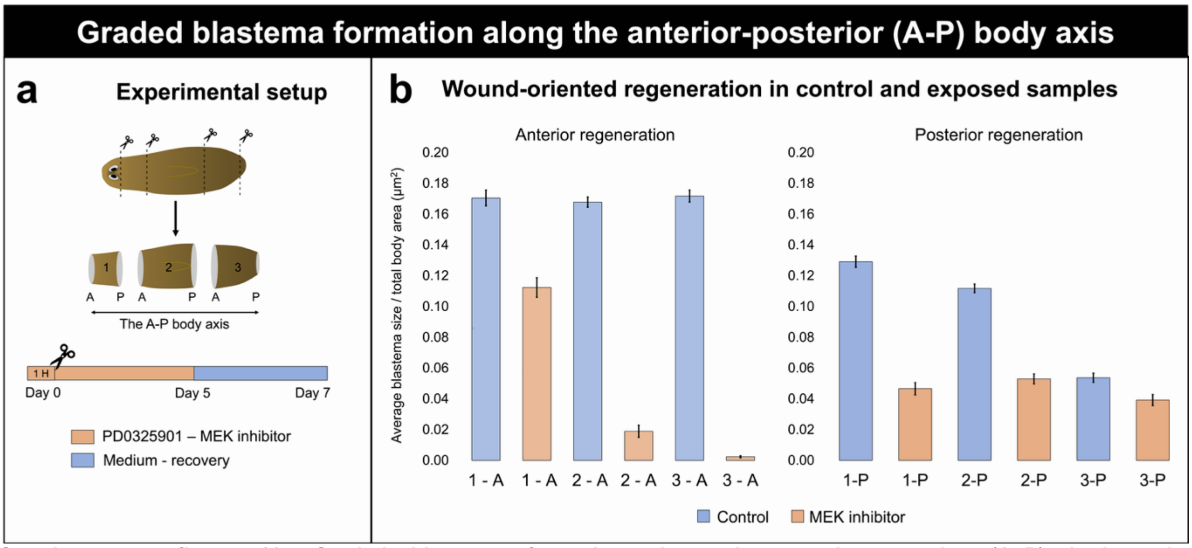

Supplementary figure 12: Graded blastema formation along the anterior–posterior (A–P) body axis. **a)** Graphical representation of the experimental setup. Animals were transversely amputated into three trunk fragments (n = 48), with both head and tail tips removed, generating fragments 1, 2, and 3. Each fragment contained an anterior (A) and posterior (P) facing wound site. MEK inhibition was achieved by treatment with PD0325901 (orange), applied 1 hour prior to amputation and maintained for 5 days during regeneration, after which animals were transferred to fresh culture medium (blue). Regenerative outcomes were assessed at 7 DPA. **b)** Quantification of graded blastema formation along the A–P body axis in control (blue) and MEK-inhibited (red) animals. The graph shows anterior and posterior blastema sizes for all three fragments: fragment 1 (1-A, 1-P), fragment 2 (2-A, 2-P), and fragment 3 (3-A, 3-P). In control animals, anterior blastema formation did not display a graded pattern along the A–P axis, whereas posterior blastema formation showed a clear decreasing gradient toward the tail. In contrast, MEK-inhibited animals exhibited the opposite pattern. Data represent mean ± SEM from three independent experiments.

Supplemental tables

Table 1: Statistical A-P differences after KD of MAPK/ERK-related targets.

KD	A-P
Smed-erk	***
Smed-mek	ns
Smed-egr-4	*

Table 2: Sequences of primers used to generate RNAi probes

Gene name	Forward primer 5'-3'	Reverse primer 5'-3'
Smed-egfr-3	GTACTGGGCAATGTTGGACCTGGC	TGACGGCCTCATGTGGGGATCATCG
Smed-mek	TTCCAGAAAGCATCCACCTT	CAGAGCTTCACTTCGCCTTC
Smed-erk	GAGAAGGTGCTTATGGAATGG	GTATCCTCGGGCTTTTAGG
Smed-egr-4	CAGACGATCTCAAA	CCATATTTGCCTGCGACATAAG
T7 sequence	GGATCCTAATACGACTCACTATAGGG	GGATCCTAATACGACTCACTATAGGG

Table 3: Sequences of qPCR primers

Target	Gene name	Forward primer 5'-3'	Reverse primer 5'-3'
GOI	*Smed-erk*	GAGCACCGGAAATAATGCTC	CCAGGAAAAAGTGGTTTGTTG
GOI	*Smed-egr-4*	GCATAAGACAATTATCATGTACGA G	CAAACCCGCGAGAAATTTTA
GOI	*Smed-mkp*	GCGCCGGACTATAATTTCAC	CGTTTGAAAGTGCCGTTG
GOI	*Smed-CuZnSOD*	TTCATGCTGTATGCGTTTTG	AACCGTGTTTACCAGGAGTTAGA
GOI	*Smed-MnSOD*	TGGGCTTGGTTAGGGTTGAA	AACGTCAAATCCTAGCAACGG
GOI	*Smed-catalase*	CCATTTAGAAATTACGAAGTCGAT G	AAGTATCTTGGGTTATGTTGAGG
GOI	*Smed-gst*	CACCAGAAGAGAAGAAAGAAGAA C	TTTTCTCCAGCGAAAAATCC
GOI	*Smed-Wnt1*	CCAAAAAGTAAGGCGGATGG	GGCTATTGAAGGAATCAGAAAGG
GOI	*Smed-Wnt11-2*	GTCAGAGGAACGACCGAAAC	CCATGAGTTCCCACTGATCC
GOI	*Smed-sFRP-1*	CGC TCT GGG GTT GAA TCT G	GTT GTC GCT GTC GAT TTG TG
GOI	*Smed-βcatenin*	CAAGAGCCCATCATCTCATTC	CAGTCCGCGTCCAACATAC
Reference	*Smed-rpl13a*	AGGTGTCCCAGCTCCTTATGA	GGCCCAATTGACAGAATTTTC
Reference	*Smed-ppia*	GCAAATGCAGGTCCAAATACA	ATGCCTTCAGCAACTTCTCC
Reference	*Smed-gapdh*	GCA AAA CAT TAT TCC GGC TTC	GCA CTG GAA CTC TAA AGG CCA
